# Proneural Transcription Factors Regulate Different Steps of Cortical Neuron Migration through Rnd-Mediated Inhibition of RhoA Signaling

**DOI:** 10.1016/j.neuron.2011.02.018

**Published:** 2011-03-24

**Authors:** Emilie Pacary, Julian Heng, Roberta Azzarelli, Philippe Riou, Diogo Castro, Mélanie Lebel-Potter, Carlos Parras, Donald M. Bell, Anne J. Ridley, Maddy Parsons, François Guillemot

**Affiliations:** 1 Division of Molecular Neurobiology, MRC National Institute for Medical Research, Mill Hill, London NW7 1AA, UK; 2 Randall Division of Cell and Molecular Biophysics, King's College London, London SE1 1UL, UK; 3 Confocal and Image Analysis Laboratory, National Institute for Medical Research, Mill Hill, London NW7 1AA, UK

## Abstract

Little is known of the intracellular machinery that controls the motility of newborn neurons. We have previously shown that the proneural protein Neurog2 promotes the migration of nascent cortical neurons by inducing the expression of the atypical Rho GTPase *Rnd2*. Here, we show that another proneural factor, Ascl1, promotes neuronal migration in the cortex through direct regulation of a second *Rnd* family member, *Rnd3*. Both Rnd2 and Rnd3 promote neuronal migration by inhibiting RhoA signaling, but they control distinct steps of the migratory process, multipolar to bipolar transition in the intermediate zone and locomotion in the cortical plate, respectively. Interestingly, these divergent functions directly result from the distinct subcellular distributions of the two Rnd proteins. Because Rnd proteins also regulate progenitor divisions and neurite outgrowth, we propose that proneural factors, through spatiotemporal regulation of Rnd proteins, integrate the process of neuronal migration with other events in the neurogenic program.

## Highlights

► The small GTPase Rnd3 is a direct target of the proneural transcription factor Ascl1 ► Rnd3 promotes cortical neuron migration by inhibiting RhoA signaling ► Rnd3 and the related protein Rnd2 have distinct roles in neuronal migration ► Rnd3 and Rnd2 have distinct subcellular distributions in cortical neurons

## Introduction

Neurons in the embryonic mammalian brain are generated in progenitor zones that line the ventricles. Soon after their birth, they undergo active cell migration to reach distant locations, where they eventually form neuronal circuits. Migration is a fundamental behavior of neurons, and migration defects during brain development result in devastating conditions, including mental retardation, autism, and epilepsy (McManus and Golden, 2005; Wegiel et al., 2010). Active research into the molecular mechanisms controlling neuronal migration has led to the discovery of extrinsic cues, receptors, and intracellular pathways that together guide neurons to their destination (Marín and Rubenstein, 2003; Sobeih and Corfas, 2002). However, much less is known of the intracellular machinery that confers a motile behavior to newly generated neurons and how this machinery is activated when neurons are born.

Transcription factors play leading roles in developmental programs that direct the differentiation of progenitor cells into mature neurons. Over the past few years, transcription factors have been shown to contribute significantly to the control of neuronal migration, with proteins such as Hoxa2 and Hoxb2 in the hindbrain and Nkx2.1 in the ventral forebrain regulating the expression of cell adhesion molecules and receptors for guidance molecules in migrating neurons (Chédotal and Rijli, 2009; Nóbrega-Pereira and Marín, 2009). However, few examples of transcription factors regulating the intrinsic migratory properties of neurons have yet been reported. In one example, Dlx1 and Dlx2 were found to promote the migration of cortical interneurons by preventing their premature differentiation through transcriptional repression of several regulators of the neuronal cytoskeleton, including microtubule-associated protein 2 (MAP2), growth-associated protein 43 (GAP43), and p-21-activated serine/threonine kinase 3 (PAK3) (Cobos et al., 2007). Recent studies have shown that Neurogenin2 (also known as Neurog2), a proneural factor with a prominent role in neurogenesis in the embryonic cortex (Nieto et al., 2001; Schuurmans et al., 2004), promotes migration in the cortex through direct induction of the expression of the small GTPase *Rnd2* and possibly other genes involved in regulating the cytoskeleton, including *RhoA*, *doublecortin*, and *p35* (Ge et al., 2006; Hand et al., 2005; Heng et al., 2008). Another proneural factor present in the embryonic cortex, Ascl1 (Britz et al., 2006) has also been shown to promote neuronal migration when overexpressed in cortical progenitors (Ge et al., 2006), although it is unclear whether this activity reflects a genuine role in cortical neuron migration and the downstream mechanisms involved are unknown.

During development of the cerebral cortex, excitatory projection neurons generated in the ventricular zone (VZ) and subventricular zone (SVZ) of the dorsal telencephalon migrate radially through the intermediate zone (IZ) to reach the superficial layers of the cortical plate (CP). Distinct phases of neuronal migration and correlated morphologies of migrating neurons can be distinguished (LoTurco and Bai, 2006). Neurons initiate migration in the VZ with a bipolar morphology, they become transiently multipolar in the SVZ and IZ, and they convert back to a bipolar morphology to enter the CP. Bipolar neurons migrate along radial glial fibers by using a mode of migration termed locomotion, which involves a reiterative succession of steps affecting different cellular domains. Neurons extend their leading process along radial glia fibers and translocate their nucleus and perinuclear region into the proximal leading process, a process known as nucleokinesis, which is followed by retraction of the trailing process, resulting in overall movement of the neuron (Marín et al., 2006). The different steps of neuronal migration involve extensive reorganization of the cytoskeleton and, not surprisingly, Rho GTPases, which control many aspects of cytoskeleton dynamics (Heasman and Ridley, 2008), have been implicated in migration of different types of neurons (Govek et al., 2005; Heasman and Ridley, 2008; Marín et al., 2006). Rac1 is required for the formation of the leading process in cortical neurons (Kawauchi et al., 2003; Konno et al., 2005), while Cdc42 is important for nuclear movements in postmitotic cerebellar granule neurons (Kholmanskikh et al., 2006), and RhoA activity is required for nucleokinesis and organization of the cytoskeleton at the rear end of migrating precerebellar neurons (Causeret et al., 2004).

Although many pathways are known to control the activity of Rho, Rac, and Cdc42 in nonneuronal cells, much less is known of how the activity of these small GTPases is controlled in migrating neurons. The atypical Rho protein Rnd3/Rho8/RhoE is an important regulator of migration of fibroblasts and tumor cells (Chardin, 2006; Guasch et al., 1998; Klein and Aplin, 2009; Nobes et al., 1998) that acts by inhibiting RhoA through stimulation of the Rho GTPase-activating protein p190RhoGAP (Wennerberg et al., 2003), and/or inhibition of the activity of ROCKI, one of the main effectors of RhoA (Riento et al., 2003). Rnd3 has been shown to induce neurite outgrowth in pheochromocytoma PC12 cells, but its role in neuronal migration has not been examined (Talens-Visconti et al., 2010). A related protein, Rnd2/Rho7/RhoN, has been shown to promote the radial migration of cortical neurons (Heng et al., 2008; Nakamura et al., 2006) and to inhibit neurite growth and induce neurite branching in PC12 cells (Fujita et al., 2002; Tanaka et al., 2006), but the mechanisms mediating Rnd2 activity in neurons remain unclear. Rnd2 and Rnd3 belong to the small Rnd family of atypical Rho proteins that lack intrinsic GTPase activity and are therefore constitutively bound to GTP (Chardin, 2006). Rnd proteins are thought to be regulated at the level of their expression, phosphorylation, and subcellular localization (Madigan et al., 2009; Riento et al., 2005a). We have previously shown that the proneural protein Neurog2 promotes the migration of nascent cortical neurons through induction of *Rnd2* expression as part of an extensive subtype-specific transcriptional program controlling cortical neurogenesis (Heng et al., 2008). In this study, we have further investigated how the cell behavior of radial migration of cortical neurons is regulated in the context of a global developmental program. We show that another proneural factor expressed in the embryonic cortex, Ascl1, promotes neuronal migration through regulation of *Rnd3*. Importantly, we demonstrate that both Rnd2 and Rnd3 inhibit RhoA signaling in cortical neurons, but that they regulate steps of migration by interfering with RhoA activity in different cell compartments. Together, our results demonstrate that proneural factors, through regulation of different Rnd proteins, integrate the process of neuronal migration with other events in the neurogenic program.

## Results

### *Rnd3* Is a Direct Transcriptional Target of Ascl1

**Figure 1:**
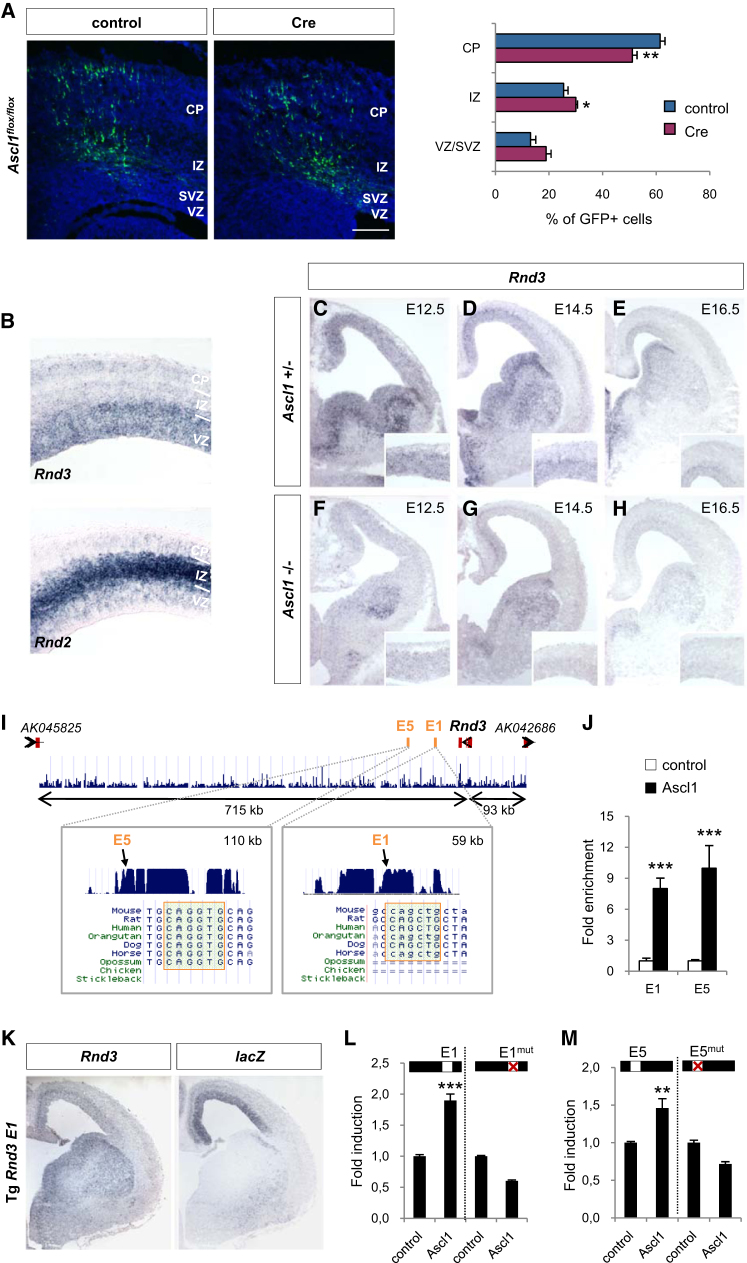
Ascl1 Directly Regulates *Rnd3* Expression in the Telencephalon (A) Distribution of electroporated (GFP^+^) cells in the cerebral cortex of embryos homozygous for a conditional null mutant allele of *Ascl1* (*Ascl1^flox/flox^*), 3 days after in utero electroporation at E14.5 of a GFP vector (control panel) or GFP and the recombinase Cre (Cre panel). TOTO-3 was used to label nuclei and subdivide the cortical wall into cortical plate (CP), intermediate zone (IZ) and subventricular zone/ventricular zone (SVZ/VZ). The graph presents the quantification of the migration of *Ascl1* mutant (Cre- and GFP-electroporated) and control (GFP-electroporated) neurons, performed by measuring the fraction of GFP^+^ cells that have reached the different zones of the cortex (VZ/SVZ, IZ, CP) 3 days after electroporation. Data are presented as the mean ± SEM from six sections prepared from three embryos obtained from two or three litters. Similar experimental design and quantification were used in subsequent experiments. Student's t test; ^∗^p < 0.05, ^∗∗^p < 0.01. Scale bar represents 150 μm. (B) Distribution of *Rnd3* and *Rnd2* transcripts in the cerebral cortex at E14.5. (C–H) Distribution of *Rnd3* transcripts in coronal sections of the developing telencephalon in control (C–E) and *Ascl1* null mutant embryos (F–H) at E12.5, E14.5, and E16.5. Cortical expression of *Rnd3* is shown at greater magnification in the insets. (I) Two evolutionarily conserved noncoding elements are located 3′ to the *Rnd3* gene and contain consensus Ascl1-binding E boxes (E1, 534 bp long and E5, 492 bp long). (J) ChIP by using an antibody against Ascl1 and chromatin prepared from E12.5 ventral telencephalon detected Ascl1 binding to the two *Rnd3* 3′ elements. For the control experiment, the same procedure was performed without antibody. The mean ± SEM of triplicate quantification from four immunoprecipitations is shown. Student's t test; ^∗∗∗^p < 0.001. (K) The *Rnd3* E1 element cloned upstream of the minimal human β-globin promoter fused to the *LacZ* reporter gene drove *LacZ* expression (*lacZ* panel, analyzed by in situ hybridization) in the dorsal telencephalon of an E14.5 transgenic embryo, in a pattern similar to that of endogenous *Rnd3* transcripts (*Rnd3* panel). (L and M) Ascl1-activated transcription from the *Rnd3* E1 (L) and E5 elements (M) in a luciferase reporter assay in P19 cells. Mutation of the Ascl1 binding motif (E1^mut^ and E5^mut^) abolished activation of the enhancers by Ascl1. n = 3, mean ± SEM; Student's t test; ^∗∗^p < 0.01, ^∗∗∗^p < 0.001. See also Figure S1.

We began this study by asking whether the proneural transcription factor Ascl1, which has been shown to enhance cell migration when overexpressed in cultured cortical cells (Ge et al., 2006), is required for neuronal migration during development of the cerebral cortex. We examined the consequence of acute *Ascl1* loss of function in the embryonic cortex by introducing an expression construct encoding the Cre recombinase in the cortex of embryos carrying a conditional mutant allele of Ascl1 (*Ascl1^flox/flox^*; Figures S1A–S1C and Supplemental Experimental Procedures). In utero electroporation of the Cre construct together with GFP to label electroporated cells in embryonic day (E) 14.5 *Ascl1^flox/flox^* mice resulted in a significant reduction of the radial migration of electroporated cells at E17.5 when compared with electroporation of only GFP (Figure 1A), demonstrating that *Ascl1* is required for proper neuronal migration in the embryonic cortex. We next asked whether *Rnd2*, which mediates the promigratory activity of *Neurog2*, is also regulating cortical neuron migration downstream of *Ascl1*. We found that *Rnd2* transcripts are normally present in the telencephalon of *Ascl1* mutant embryos, whereas they are clearly depleted in *Neurog2* mutants (Heng et al., 2008; Figure S1D), suggesting that Ascl1 does not regulate *Rnd2* expression. To identify alternative mechanisms through which Ascl1 promotes migration, we searched for candidate target genes of Ascl1 that might be involved in regulating cell migration (Gohlke et al., 2008; Figure S1E). By using gene expression microarrays, we found that *Rnd3/RhoE*, a member of the *Rnd* family of small GTP-binding proteins that also includes *Rnd2* (Chardin, 2006), was significantly downregulated in the embryonic cortex of *Ascl1* null mutant embryos and upregulated in the ventral telencephalon of embryos electroporated with an *Ascl1* expression construct (Figure S1E). *Rnd3* transcripts are found throughout embryonic development in the VZ and the CP of the cerebral cortex (Figures 1B–1E), as well as in the VZ and SVZ of the ventral telencephalon (Figures 1C–1E). *Rnd3* transcript levels were markedly reduced in embryos mutant for *Ascl1*, while they were unaffected in *Neurog2* mutant embryos (Figures 1F–1H and Figure S1D). To determine whether *Rnd3* is a direct transcriptional target of Ascl1, we performed an in silico search for putative Ascl1-regulated elements within the *Rnd3* gene locus and identified 21 distinct evolutionarily conserved regions which contained a consensus Ascl1 binding motif (CAGSTG) (Figure S1F). To evaluate Ascl1 occupancy within these putative regulatory regions, we carried out chromatin immunoprecipitation (ChIP) with an antibody against Ascl1 and chromatin prepared from embryonic telencephalon and found that Ascl1 was bound in vivo to two of these conserved elements (*Rnd3* E1, located 59 kb 3′ of the gene and *Rnd3* E5, located 110 kb 3′ of Rnd3; Figures 1I and 1J and Figure S1F). We examined the gene regulatory activity of these regions by using a transgenic mouse enhancer assay and we established that one element, *Rnd3* E1, had enhancer activity in the embryonic cortex (n = 6; Figure 1K and data not shown). We also used a luciferase reporter assay in the embryonal carcinoma cell line P19 to show that Ascl1 activates transcription from the E1 element and to a lesser extent from the E5 element and that intact Ascl1 binding motifs are required for this activity (Figures 1L and 1M). Together, these results indicate that Ascl1 regulates *Rnd3* expression in the embryonic cortex by direct regulation of the E1 enhancer and possibly other elements in the *Rnd3* locus.

### *Rnd3* Is Required for the Migration of Cortical Neurons

**Figure 2:**
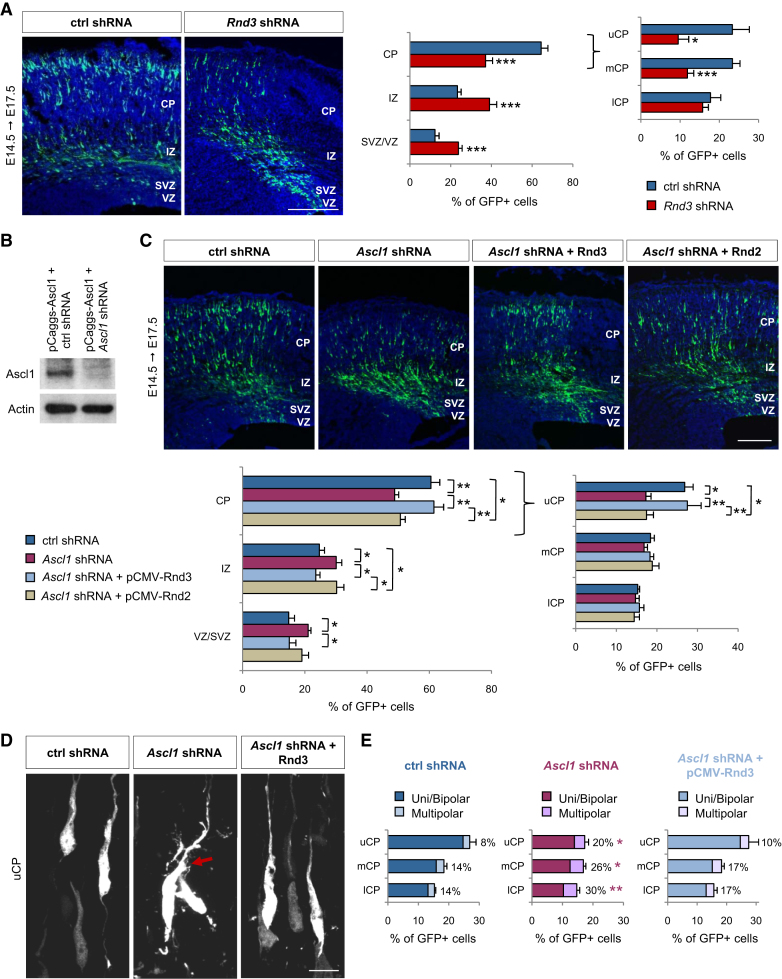
*Rnd3* Is a Major Effector of *Ascl1* in Neuronal Migration (A) Migration defects of cortical cells electroporated in utero with *Rnd3* shRNA at E14.5 and analyzed 3 days later. The graph shows the quantification of the migration of *Rnd3* shRNA and control shRNA-electroporated neurons. The CP is further subdivided into upper CP (uCP), median CP (mCP) and lower CP (lCP). Student's t test; ^∗^p < 0.05, ^∗∗∗^p < 0.001 compared to control shRNA. (B) Western blot of P19 cells cotransfected as indicated and probed with anti-Ascl1 and anti-actin antibodies to demonstrate the efficiency of *Ascl1* silencing by *Ascl1* shRNA 2 days after transfection. Expression of actin was used as a loading control. (C) Distribution of cortical cells electroporated with GFP and different constructs as indicated. *Ascl1* silencing resulted in radial migration defects that were rescued by overexpression of *Rnd3*, but not *Rnd2*. Mean ± SEM; one-way ANOVA followed by a Fisher's PLSD post hoc test; ^∗^p < 0.05, ^∗∗^p < 0.01. (D and E) *Ascl1-*silenced neurons displayed abnormal morphologies. The arrow indicates supernumerary primary processes. *Rnd3* overexpression rescued this defect. n > 500 cells from three different brains; Student's t test; ^∗^p < 0.05, ^∗∗^p < 0.01 compared to control shRNA. Scale bars represent 150 μm (A, C) and 10 μm (D). See also Figures S2–S4.

To study the function of *Rnd3* during cortical development, we used an acute loss-of-function approach by RNA interference. We designed an expression construct encoding a short hairpin RNA that efficiently and specifically knocked down *Rnd3* expression (*Rnd3* shRNA *#2*; Figures S2A, S2B, and S6A) and introduced this *Rnd3* shRNA together with EGFP expressed from the same construct by in utero electroporation in the cerebral cortex at E14.5. Examination of the electroporated brains at E17.5 (Figure 2A) revealed a marked defect in the migration of *Rnd3* shRNA-treated cells compared with control shRNA-treated cells. *Rnd3* silencing resulted after 3 days in a significant increase in the fraction of electroporated cells remaining in the VZ/SVZ (23.8 ± 1.8% of *Rnd3* shRNA-electroporated cells compared with 12.3 ± 2.0% of control shRNA-electroporated cells) and the IZ (39.1 ± 3.5% versus 23.3 ± 1.8%) and a significant decrease in the fraction of cells reaching the CP (37.1 ± 3.4% versus 64.4 ± 3.3%) and particularly the median (11.9 ± 1.6% versus 23.3 ± 1.9%) and upper parts of the CP (9.5 ± 2.7% versus 23.3 ± 4.3%; Figure 2A). To rule out a mere delay in migration, which has been observed when silencing some migration-promoting genes (Creppe et al., 2009), we electroporated the *Rnd3* shRNA at E14.5 and harvested the treated brains at postnatal day (P) 2. A significant migration defect was still observed in *Rnd3*-silenced neurons at this stage (Figure S3A), indicating that *Rnd3* is absolutely required for cortical neuron migration to proceed.

Electroporation of *Rnd3* shRNA in the cortex did not induce the death of migrating neurons or defects in radial glia processes (Figures S2C and S2E). However, it altered neural progenitor proliferation as shown by an increase in the fraction of BrdU-incorporating cells in the VZ and SVZ (Figure S2D). This suggests that *Rnd3* inhibits cell-cycle progression of cortical progenitors, a result consistent with previous studies demonstrating a role for *Rnd3* in fibroblast and tumor cell proliferation (Bektic et al., 2005; Poch et al., 2007; Villalonga et al., 2004). This finding raised the possibility that the reduced migration of *Rnd3*-silenced cells that we observed was a secondary consequence of the failure of progenitor cells to exit the cell cycle. To address this idea, we electroporated the *Rnd3* shRNA at E14.5 and we maintained electroporated brains in organotypic slice cultures for 4 days in the continuous presence of BrdU. When analyzing the migration of electroporated cells, we identified BrdU-negative cells as being already postmitotic when *Rnd3* was knocked down at the beginning of the experiment. These *Rnd3* shRNA-treated postmitotic cells presented a similar block in their migration as observed in previous experiments (Figures S2B and S2F). To confirm this finding by using another approach, we silenced *Rnd3* specifically in postmitotic cortical neurons by electroporating a vector expressing *Rnd3* shRNA in a Cre-dependent manner (Figure S2G) in the cortex of *Nex^cre^* mice, in which Cre is specifically expressed in postmitotic cortical neurons (Goebbels et al., 2006). Once again, these *Rnd3* shRNA-treated postmitotic neurons presented migration defects that were comparable to those observed with *Rnd3* shRNA alone (Figure S2G). Altogether, these results demonstrate that *Rnd3* is directly involved in the regulation of cortical neuron migration in addition to its role in cell-cycle control (E.P., unpublished data).

### *Rnd3* Is a Major Effector of *Ascl1* in Neuronal Migration

*Ascl1* promotes *Rnd3* expression in the embryonic cortex and disruption of either *Ascl1* or *Rnd3* expression leads to defective migration (Figures 1 and 2), suggesting that *Rnd3* may mediate the promigratory activity of *Ascl1* in cortical neurons. To directly address this possibility, we investigated whether forced expression of *Rnd3* in *Ascl1*-deficient neurons could rescue their migration in vivo. Remarkably, codelivery of an *Rnd3* expression construct resulted in a rescue of the migration and morphological defects of *Ascl1*-silenced neurons (Figures 2C–2E). Only 48.8 ± 1.3% of *Ascl1*-silenced neurons had reached the CP 3 days after electroporation, while 61.5 ± 3.1% of *Ascl1*-silenced cells that expressed *Rnd3* reached the CP, a proportion similar to that observed in a control experiment (60.5 ± 2.8%). Similar results were observed in rescue experiments of *Ascl1* conditional null mutant neurons with the same *Rnd3* expression construct (Figure S4A), while overexpressing *Rnd3* at the same moderate level had no apparent effect on the morphology or the migration of wild-type cortical cells (Figure S4B and data not shown). Together, these results indicate that *Rnd3* is the main effector of *Ascl1* for the promotion of radial migration.

**Figure 3:**
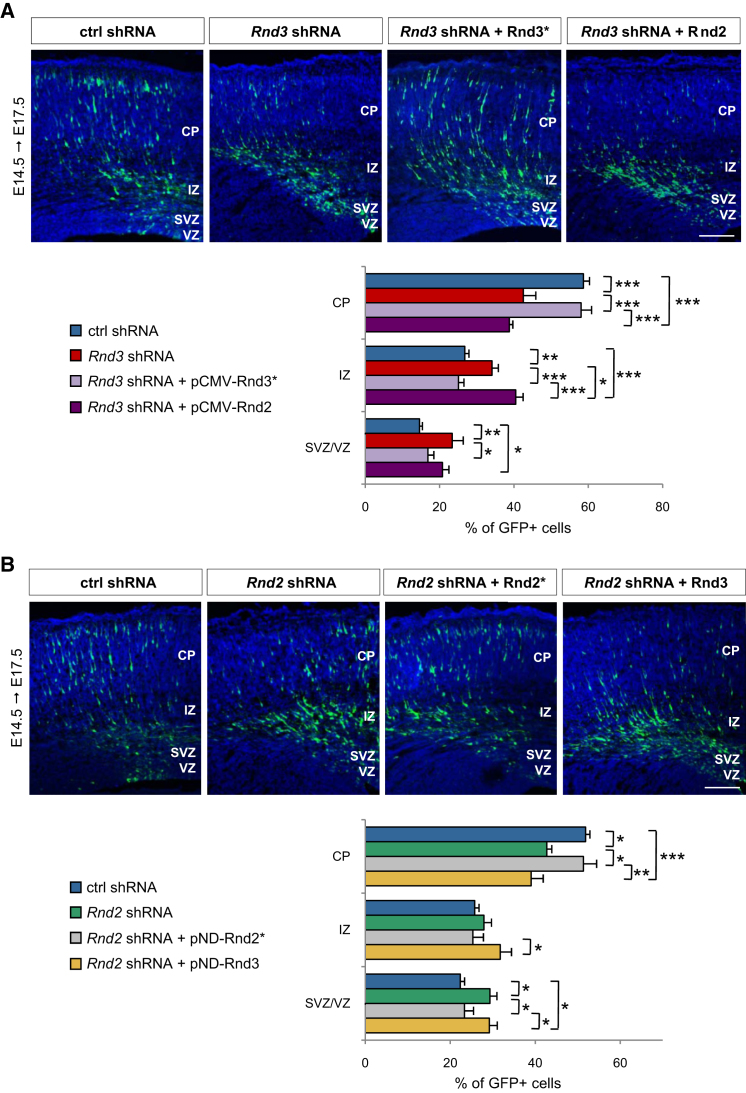
*Rnd2* and *Rnd3* Cannot Replace Each Other in Migrating Neurons (A) The radial migration defect of *Rnd3*-deficient neurons was rescued by overexpression of a shRNA-resistant version of *Rnd3* (*Rnd3^∗^*), but not by overexpression of *Rnd2*. Mean ± SEM; one-way ANOVA followed by a Fisher's PLSD post hoc test; ^∗^p < 0.05, ^∗∗^p < 0.01, ^∗∗∗^p < 0.001. (B) The migration defect of *Rnd2*-silenced neurons was rescued by overexpression of *Rnd2^∗^*, but not of *Rnd3*. Mean ± SEM; one-way ANOVA followed by a Fisher's PLSD post hoc test; ^∗^p < 0.05, ^∗∗^p < 0.01, ^∗∗∗^p < 0.001. Scale bars represent 100 μm (A, B).

This function of *Rnd3* downstream of *Ascl1* draws strong parallels with how *Neurog2* promotes neuronal migration by stimulating expression of another *Rnd* gene, *Rnd2* (Heng et al., 2008). This raised the possibility that the two *Rnd* genes have similar roles in migrating neurons, which we tested by asking whether the migration defect of *Rnd3*-silenced neurons can be rescued by *Rnd2* overexpression in vivo, and vice versa. *Rnd3* knockdown reduced the fraction of neurons reaching the CP from 58.7 ± 1.6% to 42.5 ± 3.4%. As expected, this migration defect was corrected by codelivery of an expression construct encoding an RNAi-resistant form of *Rnd3* (58.1 ± 2.8% neurons in the CP; Figure 3A). In contrast, codelivery of an expression construct for *Rnd2* did not improve the migration of *Rnd3*-silenced neurons (38.7 ± 0.9% neurons in the CP; Figure 3A). Similarly, coelectroporation of *Rnd3* expression construct with *Rnd2* shRNA failed to rescue the migration block observed in *Rnd2*-silenced neurons (Figure 3B). Taken together, these results indicate that *Rnd2* and *Rnd3* cannot be substituted for one another in cortical neurons. In agreement with these findings, *Rnd2* overexpression failed to rescue the migration defect of *Ascl1*-silenced neurons (Figure 2C) or *Ascl1* mutant neurons (Figure S4A).

### *Rnd3* and *Rnd2* Are Required during Different Phases of Migration

**Figure 4:**
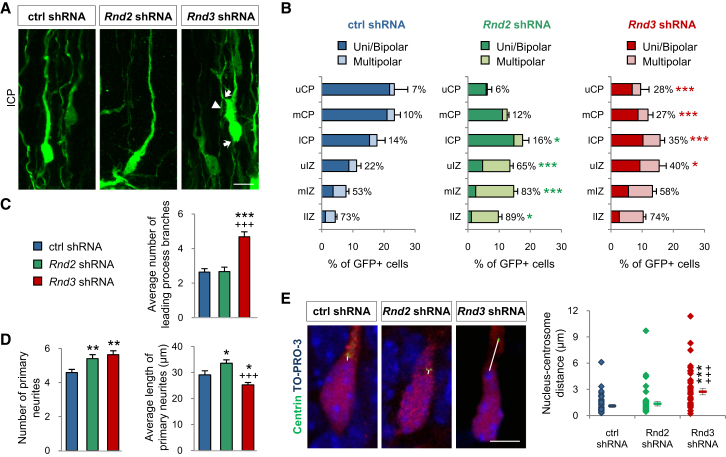
*Rnd3* and *Rnd2* Are Required during Different Phases of Migration (A) Morphology of electroporated cells in the lCP. *Rnd3*-silenced neurons displayed an enlarged leading process (arrowhead) and multiple thin processes emanating from the cell body and leading process (arrows) not seen in *Rnd2*-silenced neurons. (B) Quantification of the morphology of electroporated cells in the different zones of the cortex. The percentages represent the proportion of multipolar cells (i.e., cells exhibiting more than two primary processes) in each zone. n > 500 cells from three different brains; Student's t test; ^∗^p < 0.05, ^∗∗∗^p < 0.001 compared to control shRNA. (C) Quantification of the number of branches emanating from the leading process. Mean ± SEM; n = 19–21 cells; one-way ANOVA followed by a Fisher's PLSD post hoc test; ^∗∗∗^p < 0.001 compared to control shRNA; +++p < 0.001 compared to *Rnd2* shRNA. (D) Quantification of the number and average length of primary neurites in dissociated cortical neuron cultures established after ex vivo *Rnd2* or *Rnd3* silencing. *Rnd2* and *Rnd3* silencing both resulted in an increased number of primary processes in cultured cortical cells, but the processes were significantly longer than normal in *Rnd2*-silenced neurons and shorter in *Rnd3*-silenced neurons. The analysis was performed after two DIV by using ImageJ software. Mean ± SEM; n = 53–62 cells; one-way ANOVA followed by a Fisher's PLSD post hoc test; ^∗^p < 0.05, ^∗∗^p < 0.01 compared to control shRNA; +++p < 0.001 compared to *Rnd2* shRNA. (E) Analysis of the distance between centrosome and nucleus in cortical neurons coelectroporated with shRNA-RFP constructs and pClG2-Centrin2-Venus. The nucleus was labeled with TO-PRO-3. Mean ± SEM; n = 41 cells in all conditions; one-way ANOVA followed by a Fisher's PLSD post hoc test; ^∗∗∗^p < 0.001 compared to control shRNA; +++p < 0.001 compared to *Rnd2* shRNA. Scale bars represent 10 μm (A) and 5 μm (E). See also Figure S3 and Movie S1.

To determine the cause for the nonexchangeability of *Rnd2* and *Rnd3*, we compared their activities in neuronal migration. Silencing *Rnd2* and *Rnd3* in side-by-side knockdown experiments resulted in migration defects of similar severity (Figure S3B) and silencing the two genes simultaneously resulted in a limited worsening of the migration defect, with a small increase in cell accumulation within the VZ/SVZ and concomitant decrease in the fraction of cells reaching the CP when compared with single knockdown experiments (Figure S3B). Thus *Rnd3* and *Rnd2* are both required for the migration of cortical neurons and their individual functions are mostly distinct and nonredundant. In agreement with this interpretation, the effects of *Rnd2* and *Rnd3* silencing on the morphology of migrating neurons were drastically different (Figures 4A and 4B). *Rnd3*-silenced neurons that reached the CP presented aberrant morphologies, including a grossly enlarged leading process and multiple thin processes extending from the cell body and the leading process (Figures 4A–4C; Movie S1). An excess number of primary processes were also observed in *Rnd3*-silenced cortical neurons in culture (Figure 4D). Migration of neurons along glial fibers in the CP involves successive phases of leading process extension and cell body translocation, during which the nucleus moves toward the centrosome located in a dilation of the leading process. The enlarged proximal leading process of *Rnd3*-silenced neurons suggested that translocation of the soma into the leading process may be impaired in these cells. Indeed, the average distance between the nucleus and the centrosome in neurons of the lower CP was markedly increased in *Rnd3*-silenced neurons (2.7 ± 0.4 μm) compared with control or *Rnd2*-silenced neurons (1.1 ± 0.2 μm and 1.4 ± 0.3 μm, respectively; Figure 2E), suggesting that Rnd3 activity is required for nucleus-centrosome coupling in locomoting neurons in the CP (see also Movie S1). *Rnd2*-silenced neurons did not present this defect (Figure 4E) and had a normally shaped leading process when they reached the CP (Figure 4A), although most of them failed to leave the IZ where they accumulated with a multipolar morphology (Figure 4B; Heng et al., 2008). Together, these data suggest that *Rnd3* and *Rnd2* are required during distinct phases of migration of cortical neurons and regulate different aspects of the migratory process.

### Both Rnd3 and Rnd2 Promote Radial Migration by Inhibiting RhoA Activity

**Figure 5:**
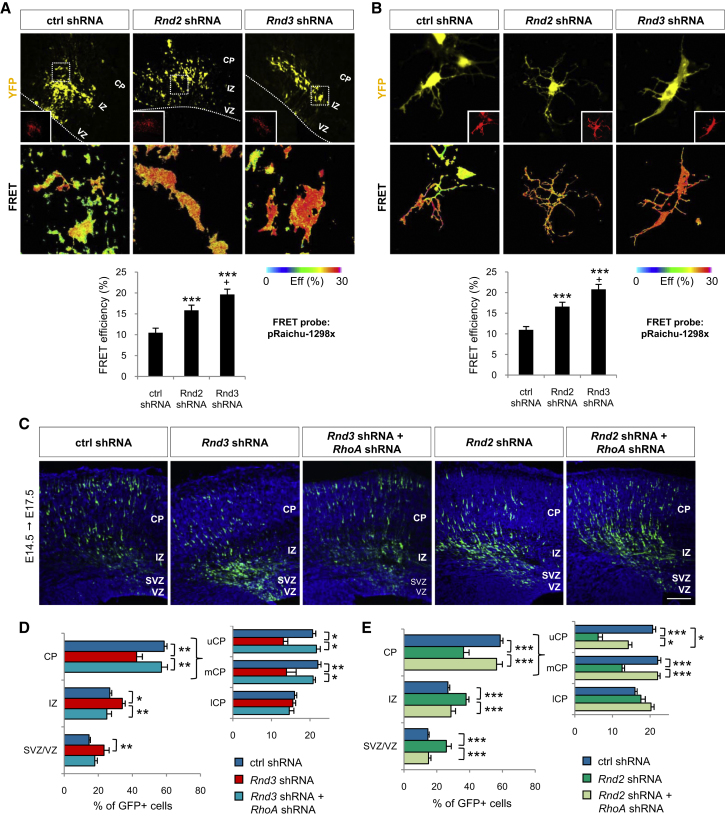
Both Rnd2 and Rnd3 Promote Neuronal Migration by Inhibiting RhoA Activity (A) FRET analysis of RhoA activity in cortical cells in vivo, 1 day after *Rnd2* or *Rnd3* knockdown. Upper panels show the YFP signal from the FRET probe 1 day after electroporation; the RFP signal in insets marks electroporated cells; lower panels show FRET efficiency in the indicated area. Mean ± SEM; t test, n = 20 cells for each condition; ^∗∗∗^p < 0.001 compared to control; +p < 0.05 compared to *Rnd2* shRNA. (B) FRET analysis of RhoA activity in dissociated cortical cells in culture, 2 days after *Rnd2* or *Rnd3* knockdown. Mean ± SEM; t test, n = 15 cells for each condition; ^∗∗∗^p < 0.001 compared to control; +p < 0.05 compared to *Rnd2* shRNA. (C–E) Coelectroporation of *RhoA* shRNA fully rescued the radial migration defects of *Rnd3*-silenced neurons and partially rescued the radial migration defects of *Rnd2*-silenced neurons. Mean ± SEM; one-way ANOVA followed by a Fisher's PLSD post hoc test; ^∗^p < 0.05, ^∗∗^p < 0.01, ^∗∗∗^p < 0.001. Scale bar represents 150 μm (C). See also Figures S5 and S6.

To understand the basis for the divergent functions of *Rnd3* and *Rnd2* in migrating neurons, we next characterized their downstream signaling pathways. Rnd3 has been shown to regulate cell morphology and migration in cultured fibroblasts and cancer cells by antagonizing RhoA (Chardin, 2006; Riento et al., 2005b). To determine whether Rnd3 also regulates RhoA signaling in the developing cortex, we measured RhoA activity in cortical cells by fluorescence resonance energy transfer (FRET) analysis. A FRET probe for RhoA (Matthews et al., 2008; Nakamura et al., 2005; Pertz et al., 2006) was electroporated with *Rnd3* shRNA or a control shRNA in the cortex of E14.5 embryos, followed by FRET analysis in brain slices 1 day after electroporation or in dissociated cortical cells after 2 days (Figures 5A and 5B). RhoA activity was detected in IZ and lower CP cells in slices as well as in dissociated cells, and this activity was significantly enhanced by *Rnd3* silencing in both settings (Figures 5A and 5B). The pathways mediating Rnd2 activity in cultured cells have not been well characterized but seem different from those operating downstream of Rnd3 (Chardin, 2006). Nevertheless, *Rnd2* shRNA electroporation in cortical cells also resulted in an increase in FRET efficiency in both slices and dissociated cells, which was less pronounced than with *Rnd3* knockdown but still significant (Figures 5A and 5B). These data therefore indicate that both Rnd2 and Rnd3 inhibit RhoA activity in migrating cortical neurons.

To determine whether antagonizing RhoA is the main mechanism by which Rnd proteins regulate radial migration, we asked whether reducing RhoA protein level in *Rnd*-silenced neurons could correct their migration defects. Coelectroporation of *Rnd3* shRNA with a *RhoA* shRNA construct that specifically and efficiently knocked down RhoA expression in P19 cells (Figure S5A and Figure 5B) fully rescued the radial migration of *Rnd3*-silenced neurons (Figures 5C and 5D). *RhoA* knockdown also rescued the migration of *Rnd2*-silenced neurons, although fewer cells coelectroporated with *RhoA* shRNA and *Rnd2* shRNA reached the upper CP than in control experiments (14.2 ± 1.6% versus 20.7 ± 2.9%; Figures 5C and 5E). Together, these experiments demonstrate that both Rnd3 and Rnd2 regulate radial migration in the cortex by inhibiting RhoA activity. In agreement with an Ascl1-Rnd3-RhoA signaling pathway promoting neuronal migration, *RhoA* knockdown also rescued the migration of *Ascl1* mutant neurons when *RhoA* shRNA was coelectroporated with Cre in *Ascl1^flox/flox^* embryos (Figure S5C).

We next used the rescue of knockdown neurons as an in vivo assay to examine the molecular mechanisms by which Rnd2 and Rnd3 regulate the RhoA signaling pathway in migrating neurons. Rnd3 can bind to the RhoA effector ROCKI and block its kinase activity (Riento et al., 2003). This interaction is disrupted by mutations of Rnd3 residues Thr173 and Val192 to arginines (Komander et al., 2008). However, *Rnd3^T173R/V192R^* was as efficient as wild-type *Rnd3* at rescuing the migration of *Rnd3*-silenced cortical cells, suggesting that Rnd3 activities in the cortex do not require interaction with ROCKI (Figures S6A, S6C, and S6D). Rnd3 can also bind to and stimulate the activity of the Rho GTPase-activating protein p190RhoGAP and this interaction is disrupted by mutation of residue T55 into valine in the effector domain of Rnd3 (Wennerberg et al., 2003). *Rnd3^T55V^* was completely inactive in the *Rnd3* knockdown rescue assay (Figures S6C and S6D), suggesting that Rnd3 antagonizes RhoA activity in migrating cortical neurons by stimulating the Rho GAP activity of p190RhoGAP. Rnd2 can also bind p190RhoGAP (Wennerberg et al., 2003) and this interaction is similarly disrupted by mutation of residue T39 in its effector domain into valine (Figure S6B). However, *Rnd2^T39V^* was as effective as wild-type *Rnd2* at rescuing the migration of *Rnd2*-silenced neurons (Figure S6F), indicating that Rnd2 activity in the cortex does not require interaction with p190RhoGAP and that Rnd2 and Rnd3 inhibit RhoA signaling via distinct mechanisms.

### Rnd3 Promotes Migration by Inhibiting RhoA-Mediated Actin Polymerization

**Figure 6:**
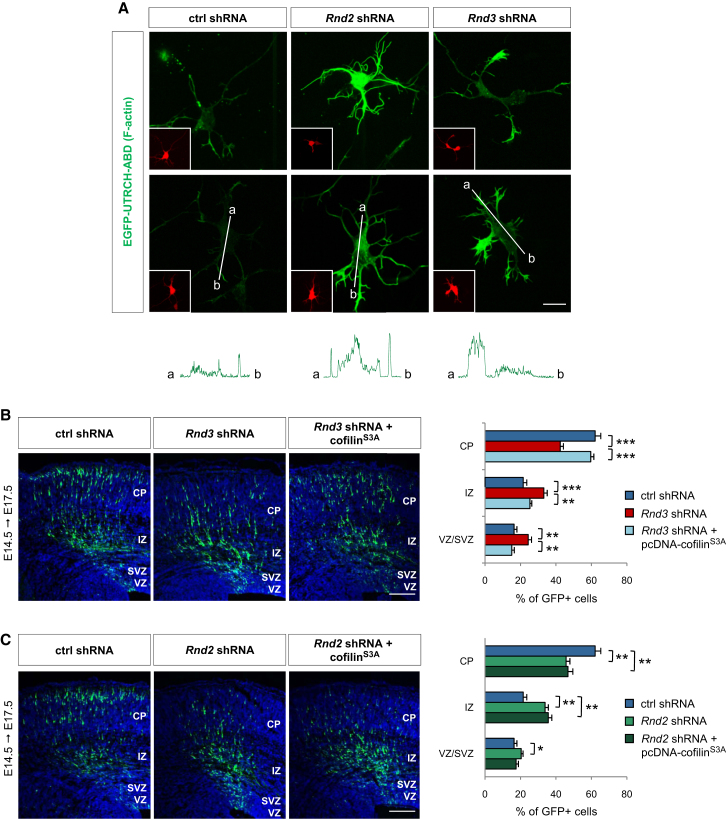
Rnd3 and Not Rnd2 Promotes Migration by Depolymerizing F-Actin (A) F-actin visualized with EGFP-UTRCH-ABD probe in dissociated cortical cells 2 days after coelectroporation of the probe and shRNAs. The RFP signals in insets mark electroporated cells. The graphs below the panels show the quantification of the green fluorescence of EGFP-UTRCH-ABD from a to b, as indicated in the panels above, by using ImageJ software. Knockdown of *Rnd3* resulted in an accumulation of F-actin in the processes of electroporated cells, while F-actin accumulated in both cell body and processes of *Rnd2* knocked-down cells. (B and C) Coelectroporation of *cofilin^S3A^*, a nonphosphorylatable form of cofilin that depolymerizes F-actin, fully rescued the migration defects of *Rnd3-*silenced neurons, but not those of *Rnd2-*silenced neurons. Mean ± SEM; one-way ANOVA followed by a Fisher's PLSD post hoc test; ^∗^p < 0.05, ^∗∗^p < 0.01, ^∗∗∗^p < 0.001. Scale bars represent 10 μm (A) and 150 μm (B, C).

As RhoA has previously been well characterized for its role in regulating the actin cytoskeleton (Govek et al., 2005; Ridley et al., 2003), we investigated whether *Rnd2* and/or *Rnd3* knockdown were altering actin dynamics in cortical neurons. We examined filamentous actin (F-actin) levels in electroporated cerebral cortical cells by coelectroporating a fluorescent F-actin probe based on the actin-binding domain of the Utrophin protein (EGFP-UTRCH-ABD). The UTRCH-ABD probe has been shown to faithfully report the presence of F-actin without altering F-actin concentrations in cells expressing the probe (Burkel et al., 2007). Knockdown of *Rnd3* resulted in a marked accumulation of F-actin in the processes of electroporated cells, while F-actin accumulated in both cell body and processes of *Rnd2* knockdown cells (Figure 6A), suggesting that both *Rnd2* and *Rnd3* regulate actin cytoskeleton organization in migrating neurons. To determine whether F-actin accumulation is responsible for the migration defects of *Rnd3*- and *Rnd2*-silenced neurons, we coelectroporated cofilin^S3A^, a nonphosphorylatable form of cofilin that constitutively depolymerizes F-actin, together with *Rnd2* or *Rnd3* shRNA. Overexpression of *cofilin^S3A^* fully rescued the migration defect of *Rnd3*-silenced neurons, thus indicating that Rnd3 promotes cortical neuron migration by inhibiting RhoA-mediated actin polymerization (Figure 6B). In contrast, *cofilin^S3A^* expression had no effect on the migration of *Rnd2*-silenced neurons demonstrating that actin remodeling does not contribute to Rnd2 migratory function and that inhibition of RhoA by Rnd2 activates another unidentified process required for neuronal migration.

### Rnd3 and Not Rnd2 Inhibits RhoA Signaling at the Plasma Membrane

**Figure 7:**
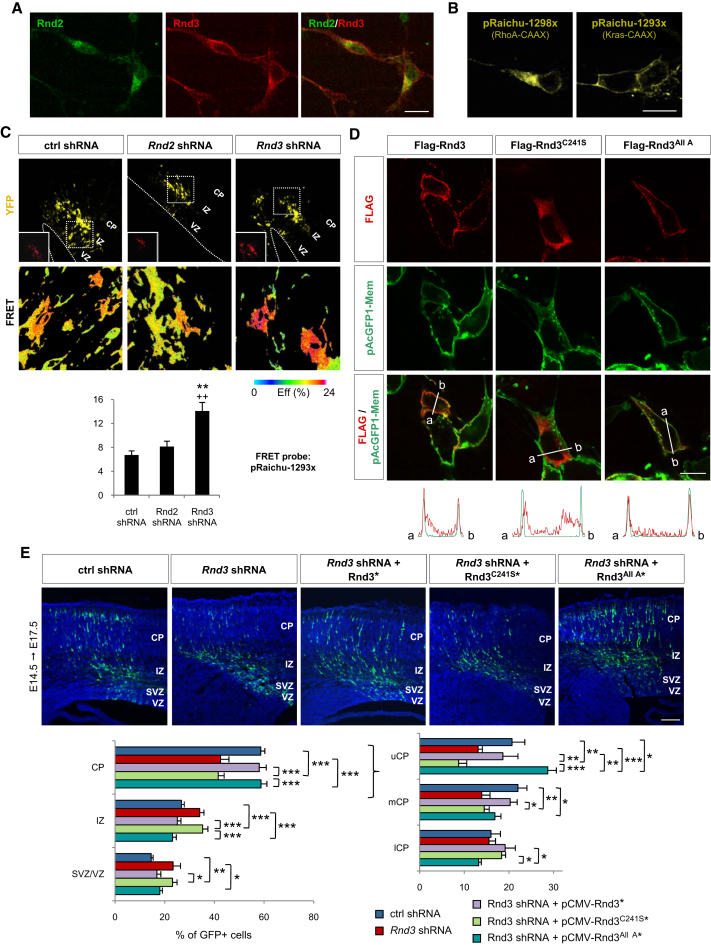
Rnd3 Activity in Migrating Neurons Requires Its Localization to the Plasma Membrane (A) Distribution of Rnd2 and Rnd3 proteins in cortical neurons harvested at E14.5 and cultured for 2 days. (B) Expression of FRET probes in dissociated cortical neurons, 2 days after electroporation. pRaichu-1298x, which carries the C-terminal region of RhoA including the CAAX box, delivers the probe preferentially to intracellular membrane compartments, whereas pRaichu-1293x, which carries the C terminus of K-Ras, delivers the probe preferentially to the plasma membrane. (C) FRET analysis of RhoA activity in cortical cells in vivo, 1 day after electroporation of pRaichu-1293x together with *Rnd2* or *Rnd3* shRNA. See Figure 5A for further details. Mean ± SEM; control shRNA, n = 15 cells; *Rnd2* shRNA, n = 12 cells; *Rnd3* shRNA, n = 16 cells; t test, ^∗∗^p < 0.01 compared to control; ++p < 0.05 compared to *Rnd2* shRNA. (D) Subcellular localization of Flag-Rnd3, Flag-Rnd3^C241S^ and Flag-Rnd3^All A^ cotransfected in HEK293 cells with pAcGFP1-Mem (Clontech) to label the plasma membrane. The graphs below the panels show the quantification of the red fluorescence marking the FLAG-tagged proteins and the green fluorescence of pAcGFP1-Mem from a to b as indicated in the panels above, by using ImageJ software. Flag-Rnd3 is present both at the plasma membrane and in the cytoplasm, Flag-Rnd3^C241S^ is absent from the plasma membrane, and Flag-Rnd3^All A^ is only present at the plasma membrane. (E) *Rnd3^C241S^*^∗^ did not rescue the migration defects of *Rnd3*-silenced neurons. Coelectroporation of *Rnd3^All A^*^∗^ with *Rnd3* shRNA increased the fraction of electroporated cells reaching the upper CP after 3 days compared to coelectroporation of *Rnd3^∗^*. Mean ± SEM; one-way ANOVA followed by a Fisher's PLSD post hoc test; ^∗^p < 0.05, ^∗∗^p < 0.01, ^∗∗∗^p < 0.001. Scale bars represent 10 μm (A, B, D) and 150 μm (E). See also Figure S7.

Our results so far have established that both Rnd3 and Rnd2 inhibit RhoA activity (Figure 5), but that they nevertheless promote migration via distinct mechanisms involving p190RhoGAP and F-actin depolymerization in the case of *Rnd3* and not *Rnd2* (Figure 6 and Figure S6). An explanation for this apparent paradox could be that Rnd2 and Rnd3 interact with RhoA in different cell compartments, because Rho GTPases have been shown to interact with different effectors and to trigger different cellular responses when located in different cell compartments (Pertz, 2010). Interestingly, the two Rnd proteins have indeed been found in distinct subcellular locations in cultured cells, with only Rnd3 localized to the plasma membrane (Armentano et al., 2006; Roberts et al., 2008). To determine whether this is also the case in cortical neurons, we examined the subcellular localization of Rnd2 and Rnd3 in dissociated cortical cells and found that Rnd3 was present in both cell processes and soma, whereas Rnd2 was only present in the soma (Figure 7A). Double labeling with antibodies against cell compartment-specific marker proteins suggested that Rnd3 is associated with the plasma membrane as well as with early endosomes and recycling endosomes, while Rnd2 appears to be associated only with early endosomes (Figure S7A, data not shown). Similar distributions of the two proteins have been previously reported in other cell types (Katoh et al., 2002; Roberts et al., 2008; Tanaka et al., 2002). To determine if these different distributions result in differential regulation of RhoA, we used a FRET probe that detects RhoA activity preferentially at the plasma membrane (Raichu-RhoA 1293x; Figure 7B; Nakamura et al., 2005). *Rnd3* knockdown resulted in a significant increase in plasma membrane-associated RhoA activity, while *Rnd2* knockdown had no significant effect (Figure 7C), suggesting that Rnd3 and Rnd2 interfere with RhoA signaling in different compartments of the migrating neurons, with only Rnd3 acting at the cell membrane.

**Figure 8:**
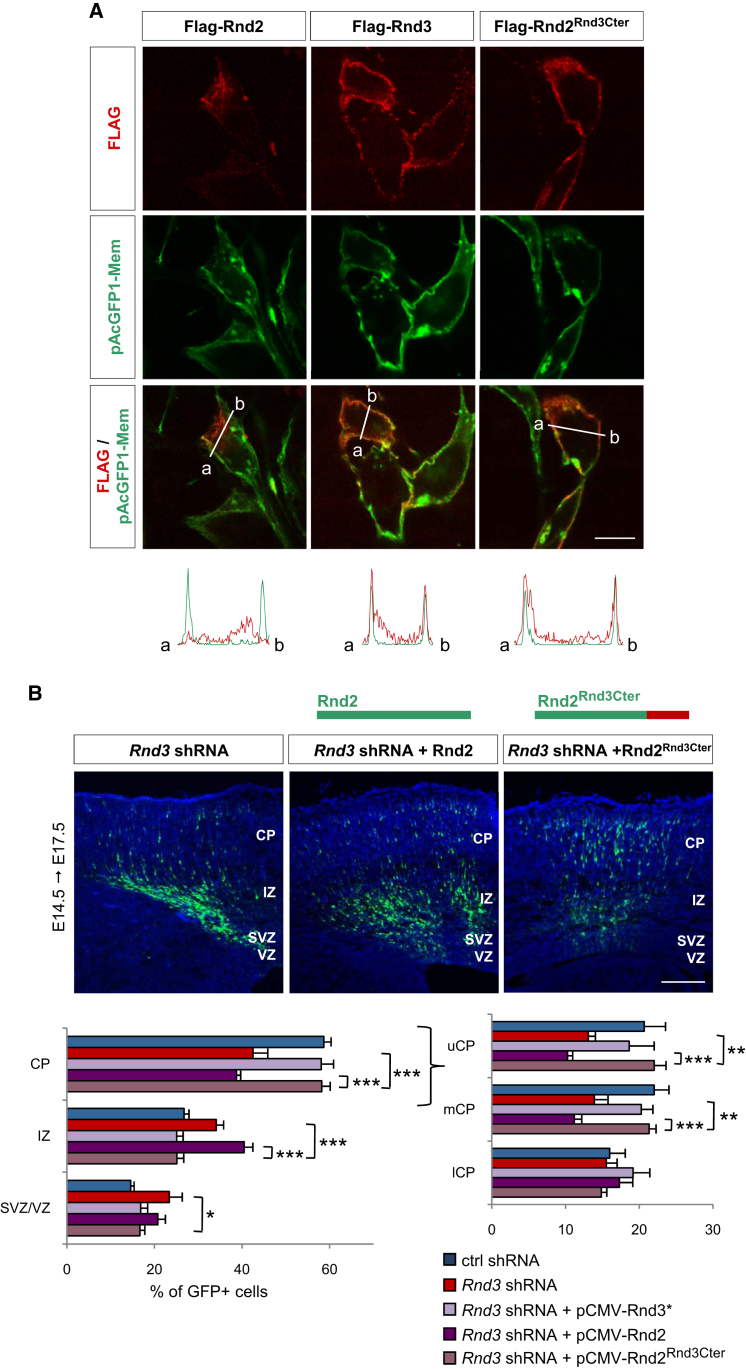
Rnd3 Function Diverges from that of Rnd2 Because of Its Localization to the Plasma Membrane (A) Subcellular localization of Flag-Rnd2, Flag-Rnd3, and a version of Rnd2 containing the C-terminal end of Rnd3 (Flag-Rnd2^Rnd3Cter^) transfected in HEK293 cells. Quantification of fluorescence below the panels is as described in Figure 7D. Flag-Rnd3 and Flag-Rnd2^Rnd3Cter^, but not Flag-Rnd2, colocalize with the plasma membrane. (B) *Rnd2^Rnd3Cter^* was as active as wild-type *Rnd3^∗^* at rescuing the migration defects of *Rnd*3-silenced neurons, while wild-type *Rnd2* had no activity in this assay. Mean ± SEM; one-way ANOVA followed by a Fisher's PLSD post hoc test; ^∗^p < 0.05, ^∗∗^p < 0.01, ^∗∗∗^p < 0.001. Scale bars represent 10 μm (A) and 150 μm (B). See also Figure S8.

We next set out to test the hypothesis that the divergent functions of *Rnd2* and *Rnd3* in neuronal migration are primarily a consequence of their distinct subcellular localizations. First, we asked whether the membrane localization of Rnd3 is essential for its activity. The membrane association of Rho proteins requires prenylation of their carboxyl-terminal CAAX motifs and is influenced by adjacent sequences (Roberts et al., 2008). Mutating the CAAX motif of Rnd3 (Rnd3^C241S^) abolished its plasma membrane association (Figure 7D) and impaired its ability to rescue the migratory activity of *Rnd3*-silenced neurons (Figure 7E and Figure S7B), thus demonstrating that membrane association is required for Rnd3 activity in migrating neurons. We next asked whether the inability of Rnd2 to replace Rnd3 in migrating neurons was due to its absence from the plasma membrane. We thus replaced the C-terminal domain of Rnd2, containing the CAAX motif and adjacent sequence, with that of Rnd3 (Figure S8A). In contrast with wild-type Rnd2, this modified Rnd2 protein (Rnd2^Rnd3Cter^) localized like Rnd3 to the plasma membrane in HEK293 cells (Figure 8A). We next examined the capacity of this plasma membrane-bound version of *Rnd2* to rescue the migration of *Rnd3*-silenced neurons. Remarkably, *Rnd2^Rnd3Cter^* was as active as *Rnd3* in this assay (Figure 8B). This demonstrates that Rnd3 owes its distinct role in neuronal migration to its localization and interaction with RhoA at the plasma membrane.

The function and localization of Rnd3 are regulated by phosphorylation of multiple serine residues in the N- and C-terminal domains of the protein (Madigan et al., 2009; Riento et al., 2005a). Phosphorylation of Rnd3 by protein kinase C promotes its translocation from the plasma membrane to internal membranes and the cytosol and reduces its ability to inhibit RhoA signaling. A nonphosphorylatable form of Rnd3, where six serine residues and one threonine residue are mutated to alanine (Rnd3^All A^), is resistant to both dissociation from the plasma membrane and attenuation of its activity by protein kinase C (Madigan et al., 2009; Figure 7D). Coelectroporation of *Rnd3^All A^* with *Rnd3* shRNA in the embryonic cortex resulted in a significantly greater fraction of electroporated cells reaching the CP after 3 days than with wild-type *Rnd3* (Figure 7E and Figure S7B). This result suggests that the membrane association and activity of Rnd3 are regulated in migrating neurons and that this determines the efficiency with which neurons migrate in the embryonic cortex.

## Discussion

In this study, we have asked how a specific cell behavior such as migration is regulated in the context of a global developmental program. We show that the two proneural factors operating in the embryonic cortex, Neurog2 and Ascl1, control distinct steps of the migratory process, multipolar to bipolar transition in the IZ and locomotion in the CP, respectively, by modulating the level of RhoA signaling in different regions of the cell, i.e., in plasma membrane versus endosomes. This exquisite level of spatiotemporal regulation is achieved through short pathways in which proneural transcription factors directly induce regulators of RhoA signaling that have restricted subcellular distributions in migrating neurons. These findings suggest that neuronal migration is not controlled by an integrated regulatory module but rather by multiple pathways that couple different steps of the migratory process with different parts of the neurogenic program.

### *Rnd3* Mediates *Ascl1* Function in Cortical Neuron Migration

We had previously shown that the proneural protein Neurog2 promotes migration in the cortex primarily through induction of the atypical Rho protein *Rnd2* (Heng et al., 2008). We now show that another proneural protein expressed in cortical progenitors, Ascl1, is also involved in the control of cortical neuron migration and that it exerts this function by inducing the expression of another member of the Rnd protein family, *Rnd3*. Remarkably, expression of *Rnd3* is sufficient to rescue the migration defect of *Ascl1*-silenced neurons, indicating that *Rnd3* is the main effector of *Ascl1* for the promotion of radial migration. It should be noted that besides its transcriptional regulation by Ascl1, our results suggest that the activity of Rnd3 is also regulated by phosphorylation in the embryonic cortex. Therefore, Rnd3 may represent a hub where a developmental program and an extrinsic signal meet to coordinate neuronal migration.

### Rnd3 and Rnd2 Regulate RhoA Activity in Partially Distinct Cell Compartments

Our characterization of the pathways through which Rnd3 and Rnd2 control migration in the embryonic cortex in vivo revealed a crucial role of inhibition of RhoA signaling. The finding that Rnd3 promotes migration of cortical neurons through inhibition of RhoA activity was not surprising, because it uses the same mechanism to regulate the migration of fibroblasts, melanoma cells, and other cell types in culture (Chardin, 2006; Guasch et al., 1998; Klein and Aplin, 2009; Riento et al., 2005b). Moreover, RhoA had been previously implicated in the control of cortical neuron migration (Hand et al., 2005; Kholmanskikh et al., 2003), although how RhoA activity is regulated in migrating neurons had remained unclear. By using an in vivo rescue assay, we provide evidence that Rnd3 antagonizes RhoA pathway in neurons through an interaction with the Rho GTPase-activating protein p190RhoGAP or with another unknown inhibitor that also requires T55 to interact with Rnd3. Other potential interactors with Rnd proteins in migrating neurons include the semaphorin receptors, Plexins, which have been implicated in cortical neuron migration (Hirschberg et al., 2010) and have been shown to be bound and regulated by Rnd proteins in neurons and other cell types (Oinuma et al., 2003; Tong et al., 2007; Uesugi et al., 2009).

The finding that Rnd2, like Rnd3, promotes migration of cortical neurons by inhibiting RhoA was more unexpected because Rnd2 does not interfere with RhoA activity in fibroblasts (Chardin, 2006; Nobes et al., 1998). The mechanism by which Rnd2 inhibits RhoA in neurons, which does not involve interaction with p190RhoGAP and is therefore different from that of Rnd3, remains to be characterized.

Although both Rnd2 and Rnd3 inhibit RhoA in migrating neurons, several lines of evidence indicate that they exert different functions: (1) the two genes cannot replace each other in shRNA rescue experiments; (2) knockdown of *Rnd2* and *Rnd3* results in very different morphological defects that appear during distinct phases of migration, and (3) the migration defect of *Rnd3*-silenced neurons, but not that of *Rnd2*-silenced neurons, can be corrected by F-actin depolymerization. We explain this apparent paradox by the fact that Rnd2 and Rnd3 have different subcellular localizations and only Rnd3 inhibits RhoA at the plasma membrane. In support of this hypothesis, we show that Rnd2 can replace Rnd3 in migrating neurons if it is targeted to the plasma membrane by replacement of its carboxyl-terminal domain with that of Rnd3. Localization of active RhoA is dynamically regulated in migrating fibroblasts (Pertz et al., 2006). The finding that Rnd3 and Rnd2 control different phases of radial migration by inhibiting RhoA in different cell compartments suggests that in cortical neurons as well, RhoA acts dynamically in different cellular domains to control different aspects of the migratory process.

Analysis of the morphological defects of knockdown neurons provides clues to the function of *Rnd3* in cortical neuron migration. *Rnd3*-silenced neurons present an increased average distance between the centrosome and the nucleus, suggesting that nucleokinesis is disrupted in these cells. *Rnd3*-silenced neurons also present a marked increase in number of leading process branches, a defect previously observed when migrating cortical neurons fail to interact properly with radial glial fibers (Elias et al., 2007; Gupta et al., 2003; Kawauchi et al., 2010; Valiente and Marín, 2010) . These results suggest a model whereby Rnd3 at the plasma membrane transduces a signal received from radial glia fibers, resulting in RhoA inhibition, F-actin depolymerization, and ultimately, stabilization and correct attachment of the leading process to radial glial fibers (Figure S8B). When this fails, the leading process may acquire an aberrant morphology and detach from radial glial fibers resulting in nucleokinesis, locomotion defects, and migration arrest.

In contrast with *Rnd3*-silenced neurons, *Rnd2*-silenced neurons appear morphologically normal when they enter the CP, indicating that *Rnd2* is not involved in the locomotion phase of migration. However, many *Rnd2*-silenced neurons remain in the IZ where they maintain a complex multipolar morphology, suggesting that *Rnd2* is required to exit the multipolar stage. The localization of Rnd2 to early endosomes suggests that it may regulate the trafficking of membrane-associated molecules that control neuronal polarization and extension of a leading process. The demonstration that Rnd2 interacts with Fnbp1/Fbp17/Rapostlin, a molecule involved in the formation of endocytic vesicles, and with Vps4-A, an important regulator of early endosome trafficking, supports this notion (Fujita et al., 2002; Kamioka et al., 2004; Tanaka et al., 2002). Together, our findings therefore suggest that through induction of *Rnd3* and *Rnd2*, *Ascl1* and *Neurog2* control successive phases of the migratory process and may thereby integrate the responses of migrating neurons to multiple extracellular signals (Figure S8B).

### A Regulatory Logic of Cortical Neuron Migration

We demonstrate that both *Ascl1* and *Neurog2* promote migration in the cerebral cortex by inhibiting RhoA activity. That proneural factors target this pathway is perhaps not surprising given the importance of Rho signaling in the regulation of cell migration (Ridley et al., 2003). More unexpected is the finding that the two proneural proteins control RhoA activity through regulation of two different target proteins with different subcellular localization. What could be the logic of this dual control of neuronal migration by proneural factors? A clue may be provided by our finding that *Rnd3* promotes not only the migration of postmitotic cortical neurons in the CP but also the cell-cycle exit of cortical progenitors in the VZ and SVZ. It has also been proposed that Rnd2 promotes dendrite branching and inhibits axon growth in differentiating neurons (Fujita et al., 2002; Negishi and Katoh, 2005; Uesugi et al., 2009). *Ascl1* and *Neurog2,* through regulation of different Rnd proteins, may thus couple particular stages of cortical neuron migration with other aspects of neurogenesis and thereby integrate the process of neuronal migration with earlier and later events in the neurogenic program.

## Experimental Procedures

### Animals

Mice were housed, bred, and treated according to the guidelines approved by the Home Office under the Animal (Scientific Procedures) Act 1986. Protocols detailing the generation and genotyping of the genetically modified mice used in this article have been described previously for *Nex^Cre^* mice (Goebbels et al., 2006) and are described in Figure S1A and Supplemental Experimental Procedures for *Ascl1^flox/flox^* mice. PCR genotyping of *Ascl1* (Guillemot et al., 1993) and *Neurog2* (Fode et al., 1998) mutant and wild-type alleles was described previously.

### In Utero Electroporation

In utero electroporation, cell counting, and statistical analyses were performed as described previously (Nguyen et al., 2006), with minor modifications as explained in the Supplemental Experimental Procedures.

### RNA In Situ Hybridization

Embryonic brains were dissected in 1 × phosphate buffered saline (PBS) and fixed overnight in 4% paraformaldehyde (PFA)/1 × PBS at 4°C. Fixed samples were cryoprotected overnight in 20% sucrose/1 × PBS at 4°C, mounted in OCT Compound (VWR International), and sectioned coronally with a cryostat (Leica). Nonradioactive RNA in situ hybridizations on frozen sections of brains were performed with digoxigenin-labeled riboprobes as described previously (Cau et al., 1997). The full-length coding sequence for mouse *Rnd3* was PCR cloned into pBluescript SK to generate an antisense probe for mouse *Rnd3*. Probes for mouse *Rnd2* (Heng et al., 2008) and *LacZ* (Seo et al., 2007) were prepared as previously described.

### Immunostaining

Immunolabelings were performed with standard protocols by using the following primary antibodies: mouse anti-Ascl1 (1/200, gift from D.J. Anderson), rat anti-BrdU (1/1000, AbD Serotec), rabbit anti-Caspase-3 (1/1000, R&D Systems), goat anti-EEA1 (1/50, Santa Cruz), chicken anti-GFP (1/700, Millipore), mouse anti-Flag (1/250, Sigma), mouse anti-LAMP1 (1/100, Developmental Studies Hybridoma Bank), mouse anti N-cadherin (1/200, BD Biosciences), mouse anti-Nestin (1/100, Millipore), mouse anti-Rab7 (1/500, Abcam), rabbit anti-Rnd2 (1/50, Santa Cruz), mouse anti-Rnd3 (1/500, Abcam), rabbit anti-Rnd3 (1/50, Abcam), and mouse anti-transferrin receptor (1/100, Zymed). Cells or sections were then incubated with appropriate fluorescent secondary antibodies. Pretreatment with 2 N HCl for 30 min at 37°C prior to preincubation with primary antibody was performed to detect BrdU.

### Fluorescence Resonance Energy Transfer

For in vivo FRET analysis, cortices were coelectroporated in utero at E14.5 with the FRET probes for RhoA pRaichu-1298x or pRaichu-1293x (0.25 μg/μl) and control shRNA-RFP, *Rnd2* shRNA-RFP, or *Rnd3* shRNA-RFP (1 μg/μl). RhoA FRET efficiency was analyzed 1 day later in fixed brain sections. For FRET analysis in dissociated cortical cells, cortices were coelectroporated ex vivo at E14.5 with the same constructs and sliced with a vibratome immediately after electroporation and sections were cultured overnight. The next day, the electroporated slices were dissociated, single cells were plated, and RhoA FRET efficiency was analyzed after 2 DIV. FRET analysis is detailed in the Supplemental Experimental Procedures.

Luciferase assays and ChIP experiments were performed as previously described with minor modifications (Castro et al., 2006) and are detailed in the Supplemental Experimental Procedures, as are plasmid constructs, cell culture, western blotting, and immunoprecipitation.

## Supplemental Information

Document S1. Eight Figures and Supplemental Experimental Procedures

Movie S1. Ex Vivo Electroporation E14.5 + 1 DIV Control shRNA
