# Supplementary material for: Proneural Transcription Factors Regulate Different Steps of Cortical Neuron Migration through Rnd-Mediated Inhibition of RhoA Signaling
Source: Neuron. 2011 Mar 24;69-540(6-7):1069–84. doi: 10.1016/j.neuron.2011.02.018 (PMC3383999; doi:10.1016/j.neuron.2011.02.018)
Supplement: Document S1. Eight Figures and Supplemental Experimental Procedures [file mmc1.pdf]

Neuron, Volume *69*

Supplemental Information

Proneural Transcription Factors Regulate Different Steps of Cortical Neuron Migration through Rnd-Mediated Inhibition of RhoA Signaling

Emilie Pacary, Julian Heng, Roberta Azzarelli, Philippe Riou, Diogo Castro, Mélanie Lebel-Potter, Carlos Parras, Donald M. Bell, Anne J. Ridley, Maddy Parsons, and François Guillemot

Figure S1

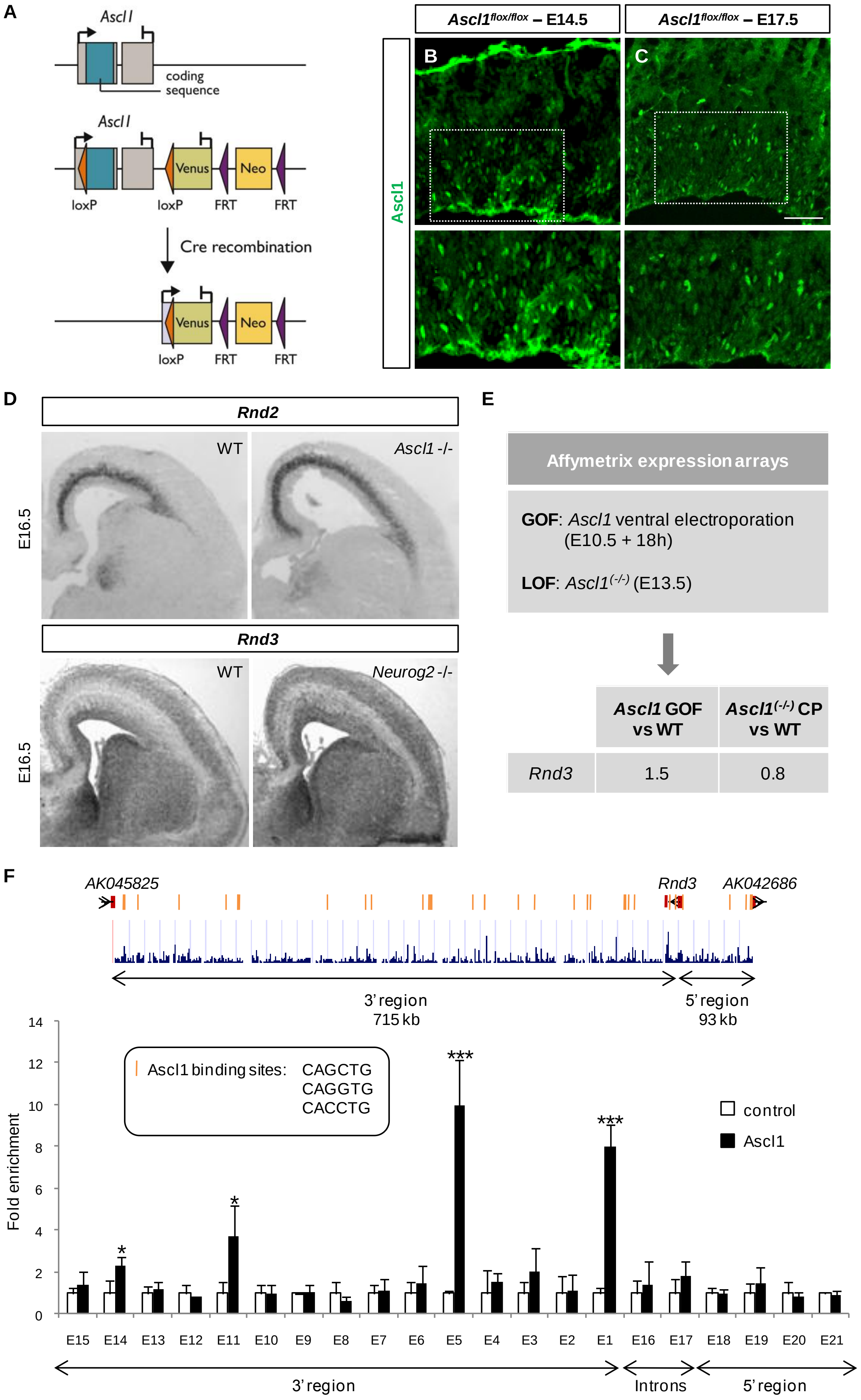
A
Ascl1
coding sequence
Ascl1
Venus
Neo
loxP
loxP
FRT
FRT
Cre recombination
Venus
Neo
loxP
FRT
FRT
Ascl1flox/flox – E14.5
Ascl1flox/flox – E17.5
B
C
Ascl1
D
Rnd2
WT
Ascl1 -/-
E16.5
Rnd3
WT
Neurog2 -/-
E16.5
E
Affymetrix expression arrays
GOF: Ascl1 ventral electroporation (E10.5 + 18h)
LOF: Ascl1(-/-) (E13.5)
Ascl1 GOF vs WT
Ascl1(-/-) CP vs WT
Rnd3
1.5
0.8
F
AK045825
Rnd3
AK042686
3' region 715 kb
5' region 93 kb
Ascl1 binding sites: CAGCTG CAGGTG CACCTG
control
Ascl1
Fold enrichment
0
2
4
6
8
10
12
14
*
*
***
***
E15
E14
E13
E12
E11
E10
E9
E8
E7
E6
E5
E4
E3
E2
E1
E16
E17
E18
E19
E20
E21
3' region
Introns
5' region

Figure S2

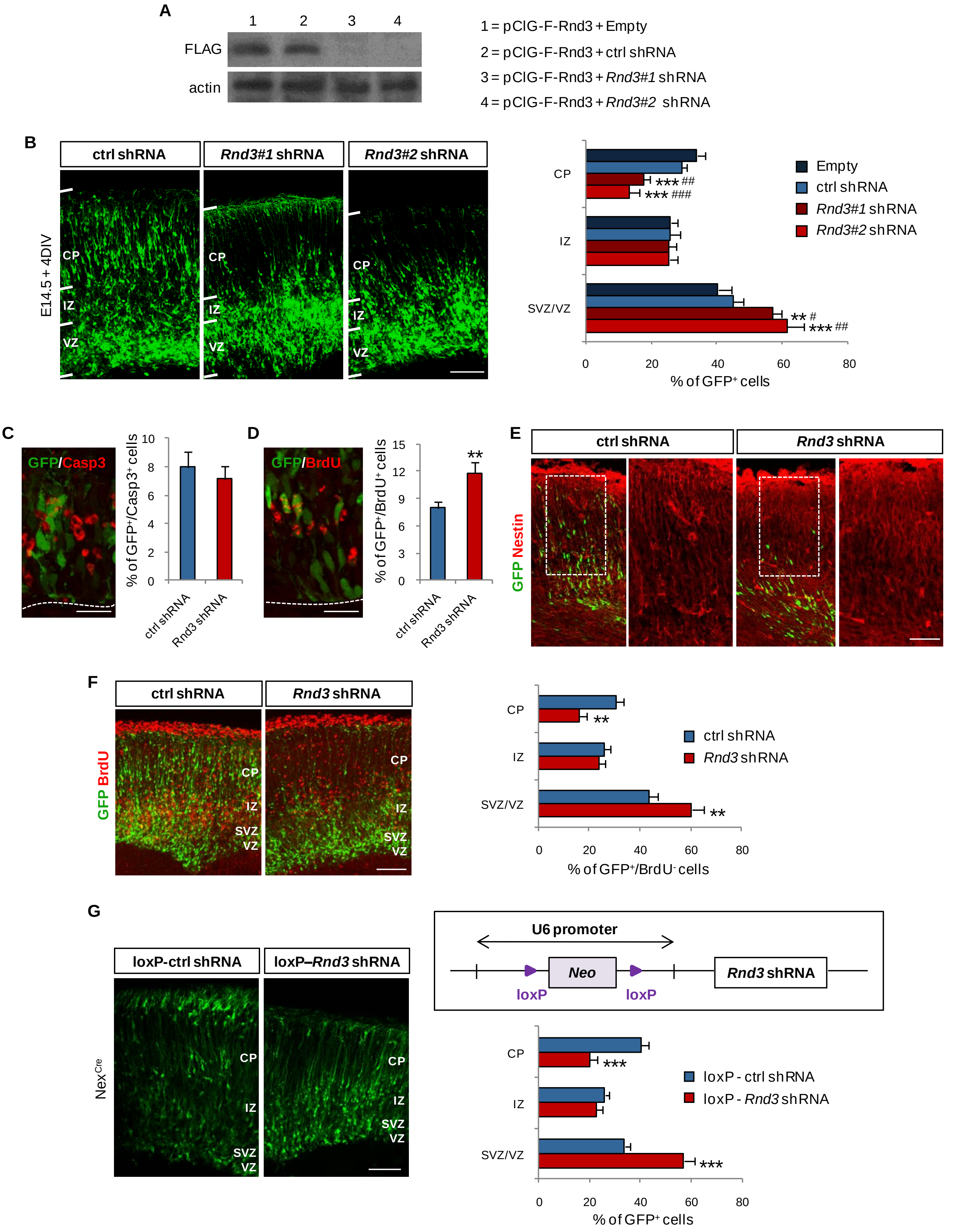

A
1
2
3
4
FLAG
actin
1 = pCIG-F-Rnd3 + Empty
2 = pCIG-F-Rnd3 + ctrl shRNA
3 = pCIG-F-Rnd3 + Rnd3#1 shRNA
4 = pCIG-F-Rnd3 + Rnd3#2 shRNA
B
ctrl shRNA
Rnd3#1 shRNA
Rnd3#2 shRNA
E14.5 + 4DIV
CP
IZ
VZ
Empty
ctrl shRNA
Rnd3#1 shRNA
Rnd3#2 shRNA
SVZ/VZ
% of GFP+ cells
C
GFP/Casp3
% of GFP+/Casp3+ cells
D
GFP/BrdU
% of GFP+/BrdU+ cells
E
ctrl shRNA
Rnd3 shRNA
GFP Nestin
F
GFP BrdU
SVZ
% of GFP+/BrdU- cells
G
loxP-ctrl shRNA
loxP–Rnd3 shRNA
NexCre
U6 promoter
Neo
loxP
Rnd3 shRNA
loxP - ctrl shRNA
loxP - Rnd3 shRNA

Figure S3

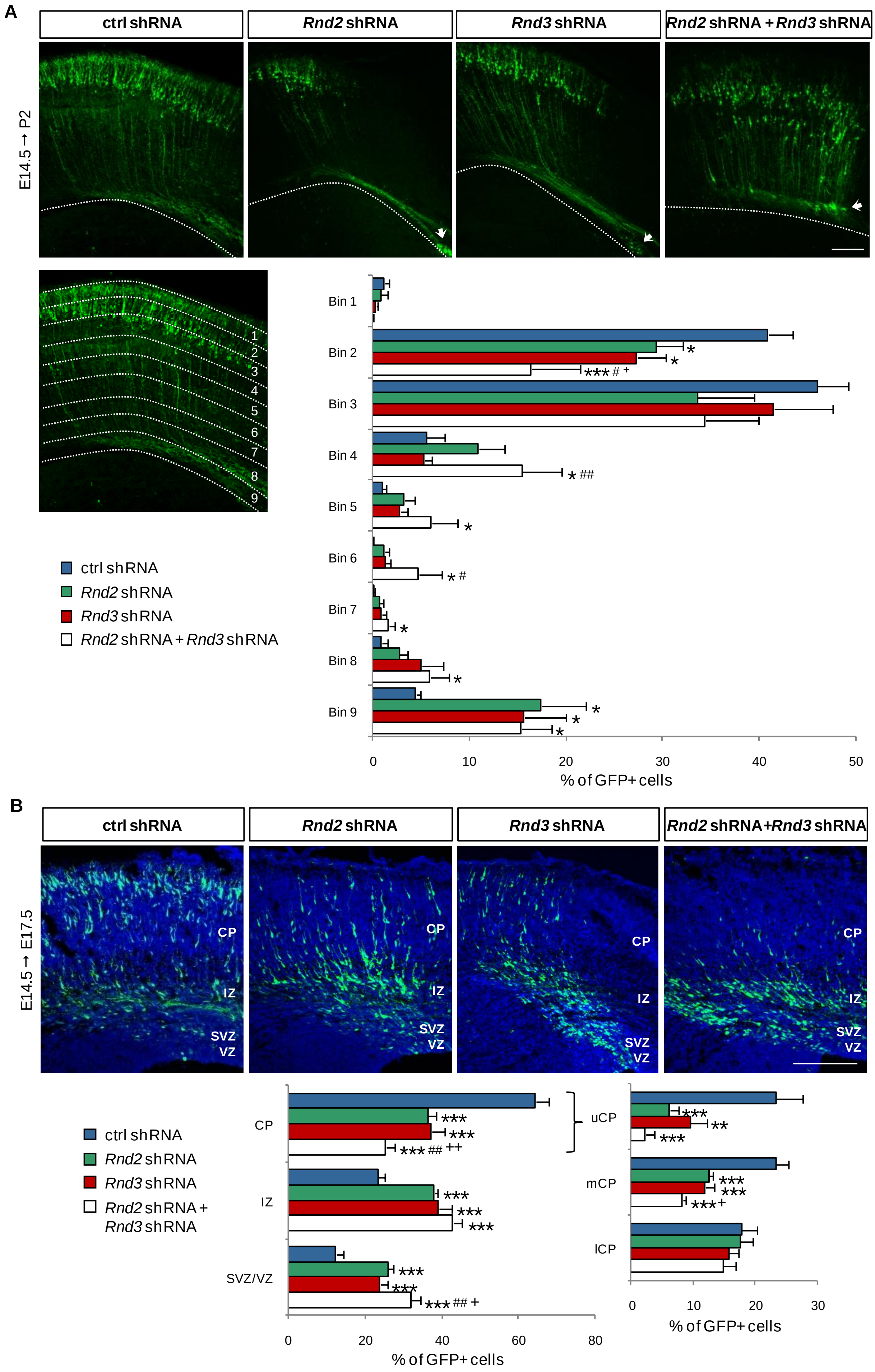

A
ctrl shRNA
Rnd2 shRNA
Rnd3 shRNA
Rnd2 shRNA + Rnd3 shRNA
E14.5 → P2
1
2
3
4
5
6
7
8
9
Bin 1
Bin 2
Bin 3
Bin 4
Bin 5
Bin 6
Bin 7
Bin 8
Bin 9
0
10
20
30
40
50
% of GFP+ cells
ctrl shRNA
Rnd2 shRNA
Rnd3 shRNA
Rnd2 shRNA + Rnd3 shRNA
B
ctrl shRNA
Rnd2 shRNA
Rnd3 shRNA
Rnd2 shRNA+Rnd3 shRNA
E14.5 → E17.5
CP
IZ
SVZ
VZ
ctrl shRNA
Rnd2 shRNA
Rnd3 shRNA
Rnd2 shRNA + Rnd3 shRNA
CP
IZ
SVZ/VZ
0
20
40
60
80
% of GFP+ cells
uCP
mCP
lCP
0
10
20
30
% of GFP+ cells

Figure S4

A

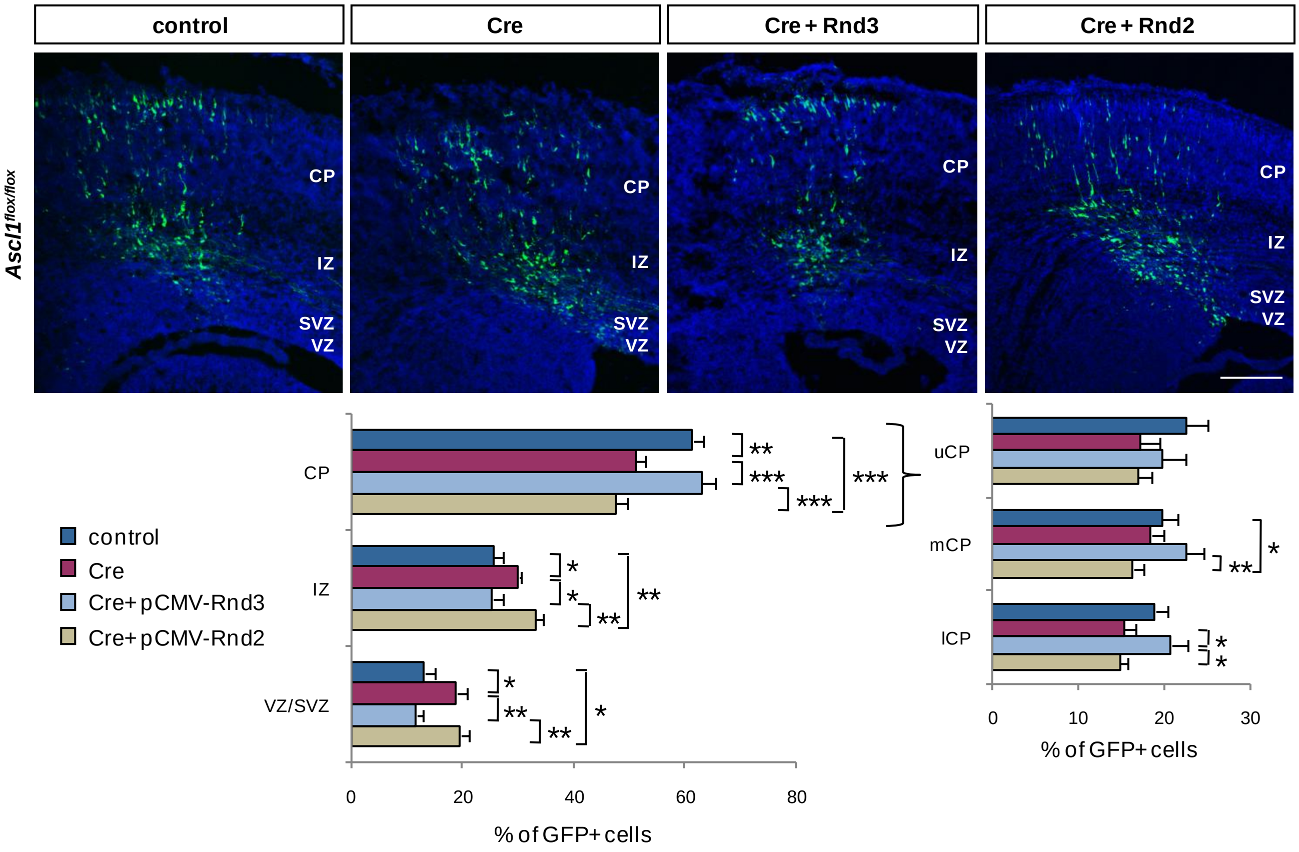
control
Cre
Cre + Rnd3
Cre + Rnd2
Ascl1flox/flox
CP
IZ
SVZ
VZ
control
Cre
Cre+pCMV-Rnd3
Cre+pCMV-Rnd2
CP
IZ
VZ/SVZ
uCP
mCP
lCP
% of GFP+ cells

B

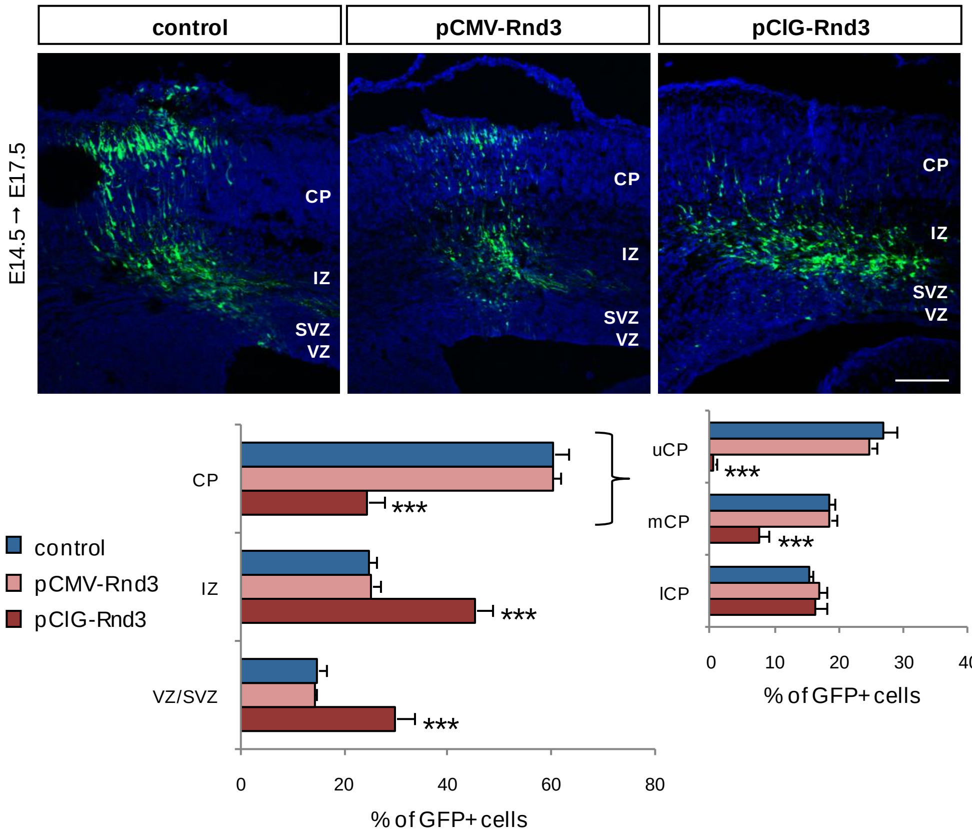
control
pCMV-Rnd3
pCIG-Rnd3
E14.5 → E17.5
CP
IZ
SVZ
VZ
control
pCMV-Rnd3
pCIG-Rnd3
CP
IZ
VZ/SVZ
uCP
mCP
lCP
% of GFP+ cells

Figure S5

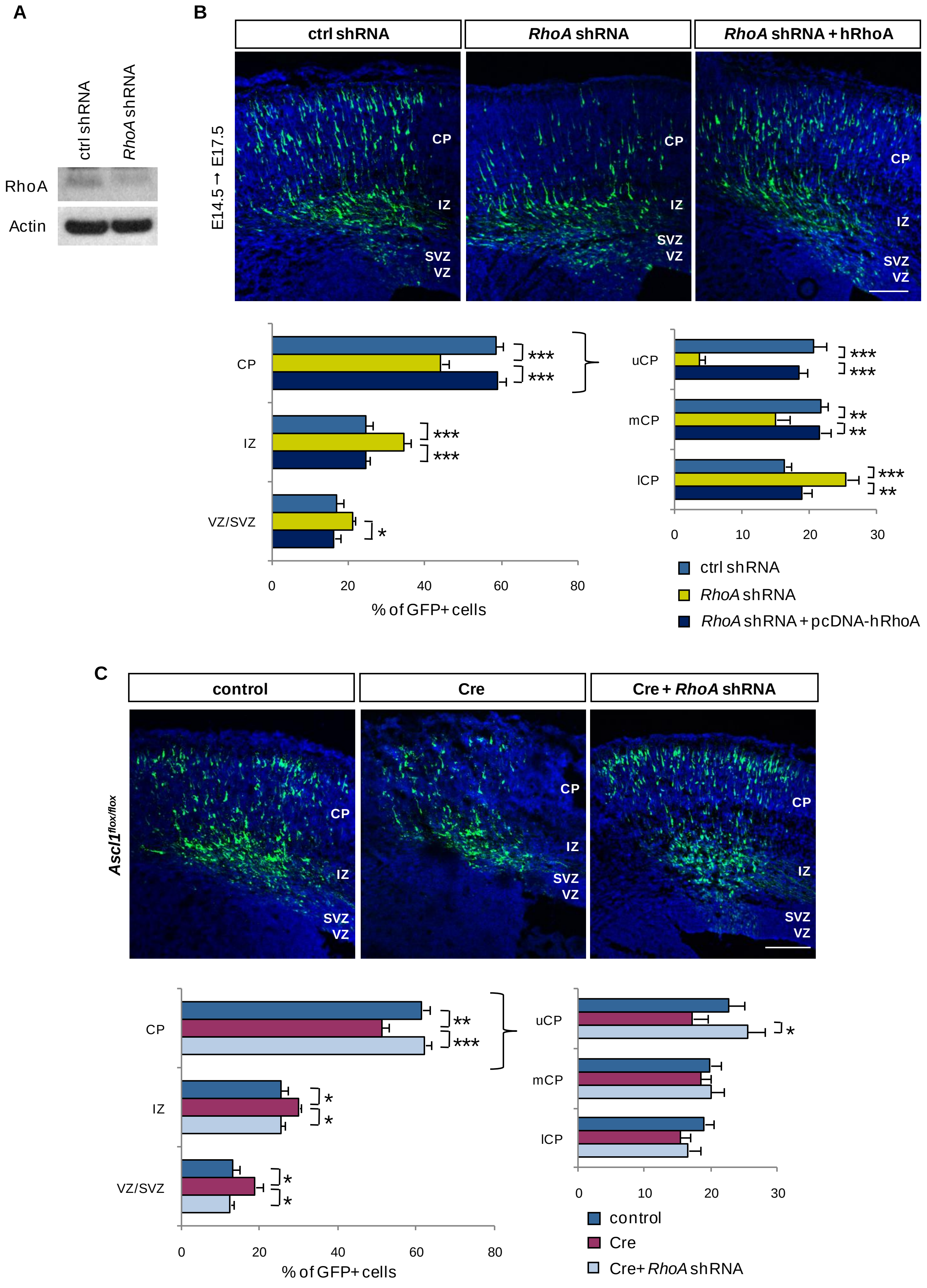

A
ctrl shRNA
RhoA shRNA
RhoA
Actin
B
ctrl shRNA
RhoA shRNA
RhoA shRNA + hRhoA
E14.5 → E17.5
CP
IZ
SVZ
VZ
CP
IZ
VZ/SVZ
% of GFP+ cells
uCP
mCP
lCP
ctrl shRNA
RhoA shRNA
RhoA shRNA + pcDNA-hRhoA
C
control
Cre
Cre + RhoA shRNA
Ascl1 flox/flox
CP
IZ
SVZ
VZ
CP
IZ
VZ/SVZ
% of GFP+ cells
uCP
mCP
lCP
control
Cre
Cre+ RhoA shRNA

Figure S6

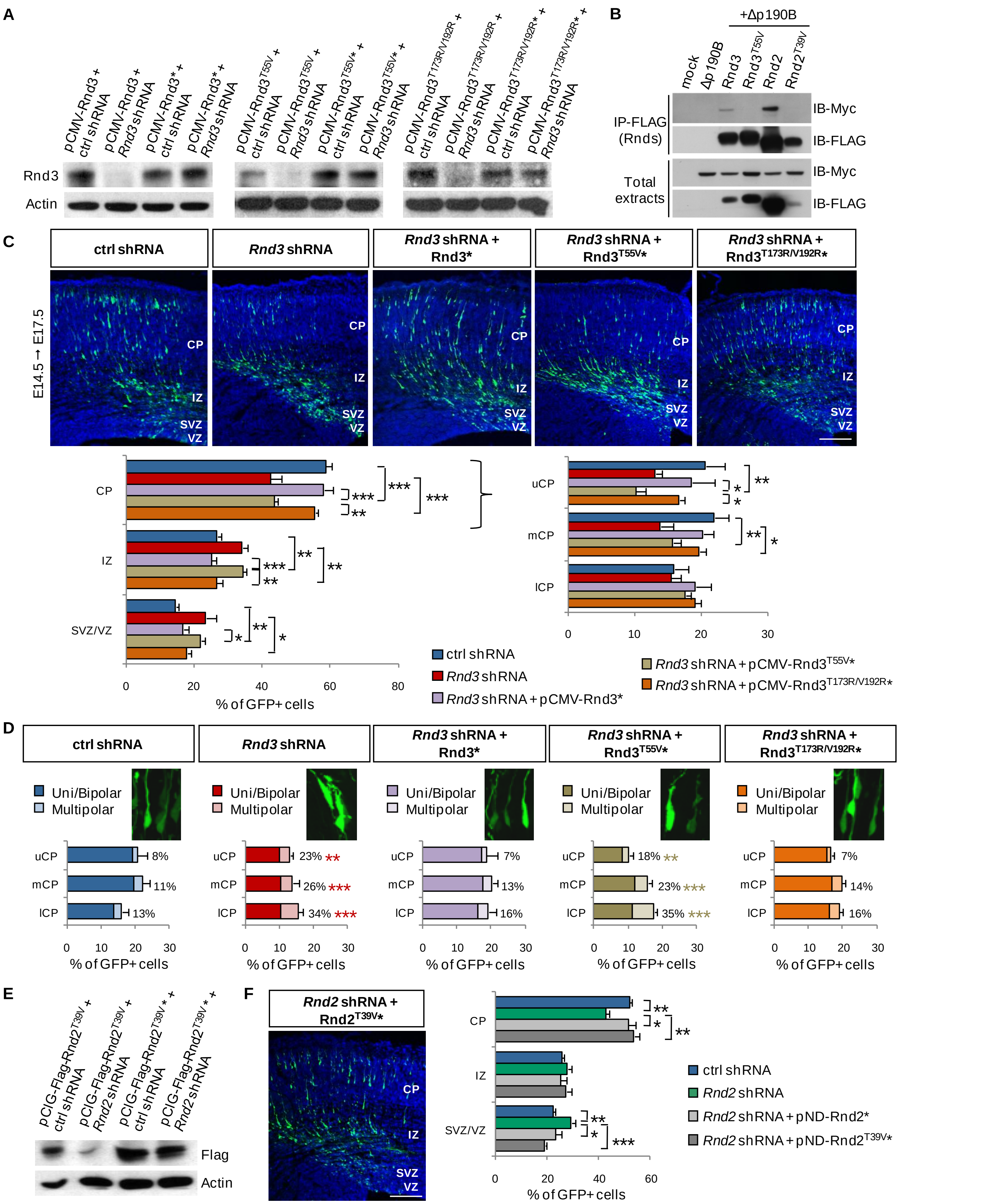

A
pCMV-Rnd3 + ctrl shRNA
pCMV-Rnd3 + Rnd3 shRNA
pCMV-Rnd3* + ctrl shRNA
pCMV-Rnd3* + Rnd3 shRNA
pCMV-Rnd3 T55V + ctrl shRNA
pCMV-Rnd3 T55V + Rnd3 shRNA
pCMV-Rnd3 T55V* + ctrl shRNA
pCMV-Rnd3 T55V* + Rnd3 shRNA
pCMV-Rnd3 T173R/V192R + ctrl shRNA
pCMV-Rnd3 T173R/V192R + Rnd3 shRNA
pCMV-Rnd3 T173R/V192R* + ctrl shRNA
pCMV-Rnd3 T173R/V192R* + Rnd3 shRNA
Rnd3
Actin
B
+Δp190B
mock
Δp190B
Rnd3
Rnd3 T55V
Rnd2
Rnd2 T39V
IP-FLAG (Rnds)
Total extracts
IB-Myc
IB-FLAG
IB-Myc
IB-FLAG
C
ctrl shRNA
Rnd3 shRNA
Rnd3 shRNA + Rnd3*
Rnd3 shRNA + Rnd3 T55V*
Rnd3 shRNA + Rnd3 T173R/V192R*
E14.5 → E17.5
CP
IZ
SVZ
VZ
CP
IZ
SVZ/VZ
***
***
***
**
***
**
**
**
**
*
*
0
20
40
60
80
% of GFP+ cells
uCP
mCP
lCP
**
*
*
**
*
0
10
20
30
ctrl shRNA
Rnd3 shRNA
Rnd3 shRNA + pCMV-Rnd3*
Rnd3 shRNA + pCMV-Rnd3 T55V*
Rnd3 shRNA + pCMV-Rnd3 T173R/V192R*
D
ctrl shRNA
Rnd3 shRNA
Rnd3 shRNA + Rnd3*
Rnd3 shRNA + Rnd3 T55V*
Rnd3 shRNA + Rnd3 T173R/V192R*
Uni/Bipolar
Multipolar
uCP
mCP
lCP
8%
11%
13%
23% **
26% ***
34% ***
7%
13%
16%
18% **
23% ***
35% ***
7%
14%
16%
0
10
20
30
% of GFP+ cells
E
pCIG-Flag-Rnd2 T39V + ctrl shRNA
pCIG-Flag-Rnd2 T39V + Rnd2 shRNA
pCIG-Flag-Rnd2 T39V* + ctrl shRNA
pCIG-Flag-Rnd2 T39V* + Rnd2 shRNA
Flag
Actin
F
Rnd2 shRNA + Rnd2 T39V*
CP
IZ
SVZ
VZ
CP
IZ
SVZ/VZ
**
*
**
**
*
***
0
20
40
60
% of GFP+ cells
ctrl shRNA
Rnd2 shRNA
Rnd2 shRNA + pND-Rnd2*
Rnd2 shRNA + pND-Rnd2 T39V*

Figure S7

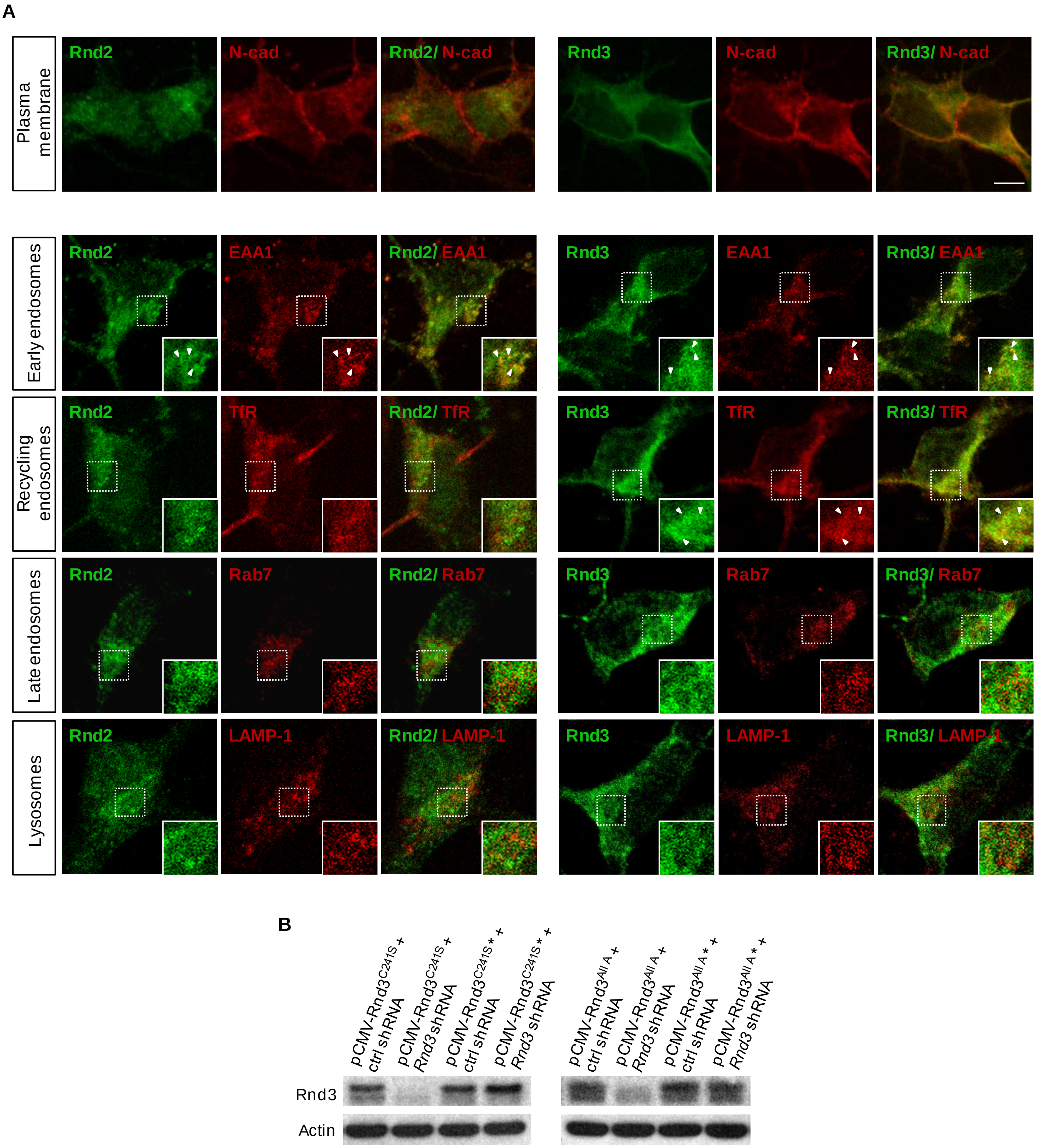
A
Plasma membrane
Early endosomes
Recycling endosomes
Late endosomes
Lysosomes
Rnd2
N-cad
Rnd2/ N-cad
Rnd3
Rnd3/ N-cad
EAA1
Rnd2/ EAA1
Rnd3/ EAA1
TfR
Rnd2/ TfR
Rnd3/ TfR
Rab7
Rnd2/ Rab7
Rnd3/ Rab7
LAMP-1
Rnd2/ LAMP-1
Rnd3/ LAMP-1
B
pCMV-Rnd3C241S + ctrl shRNA
pCMV-Rnd3C241S + Rnd3 shRNA
pCMV-Rnd3C241S* + ctrl shRNA
pCMV-Rnd3C241S* + Rnd3 shRNA
pCMV-Rnd3All A + ctrl shRNA
pCMV-Rnd3All A + Rnd3 shRNA
pCMV-Rnd3All A* + ctrl shRNA
pCMV-Rnd3All A* + Rnd3 shRNA
Rnd3
Actin

A

```
               ....|....| ....|....| ....|....| ....|....| ....|....|
                        10         20         30         40         50
Rnd2 mouse     ---------- -----ME-GQ SGRCKIVVVG DAECGKTALL QVFAKDAYPG
Rnd3 mouse     MKERRASQKL SSKSIMDPNQ NVKCKIVVVG DSQCGKTALL HVFAKDCFPE
               ....|....| ....|....| ....|....| ....|....| ....|....|
                        60         70         80         90        100
Rnd2 mouse     SYVPTVFENY TASFEIDKRR IELNMWDTSG SSYYDNVRPL AYPDSDAVLI
Rnd3 mouse     NYVPTVFENY TASFEIDTQR IELSLWDTSG SPYYDNVRPL SYPDSDAVLI
               ....|....| ....|....| ....|....| ....|....| ....|....|
                       110        120        130        140        150
Rnd2 mouse     CFDISRPETL DSVLKKWQGE TQEFCPNAKV VLVGCKLDMR TDLATLRELS
Rnd3 mouse     CFDISRPETL DSVLKKWKGE IQEFCPNTKM LLVGCKSDLR TDVSTLVELS
               ....|....| ....|....| ....|....| ....|....| ....|....|
                       160        170        180        190        200
Rnd2 mouse     KQRLIPVTHE QGTVLAKQVG AVSYVECSSR SSERSVRDVF HVATVASLGR
Rnd3 mouse     NHRQTPVSYD QGANMAKQIG AATYIECSAL QSENSVRDIF HVATLACVNK
               ....|....| ....|....| ....|....| ....|....| ....
                       210        220        230        240
Rnd2 mouse     GHRQLRRTDS RRGLQRSTQL SGRPDRGNEG -EMHKDRAKS CNLM
Rnd3 mouse     TNKNVKRNKS QRATKRISHM PSRPELSAVA TDLRKDKAKS CTVM
```

Rnd2^{Rnd3Cter}:

```
MEGQSGRCKIVVVGDAECGKTALLQVFAKDAYPGSYVPTVFENYTASFEIDKRRIELNMWDTSGSSYYD
NVRPLAYPDSDAVLICFDISRPETLDSVLKKWQGETQEFCPNAKVVLVGCKLDMRTDLATLRELSKQRL
IPVTHEQGTVLAKQVGAVSYVECSSRSSERSVRDVFHVATVACVNKTNKNVKRNKSQRATKRISHMPSR
PELSAVATDLRKDKAKSCTVM
```

B

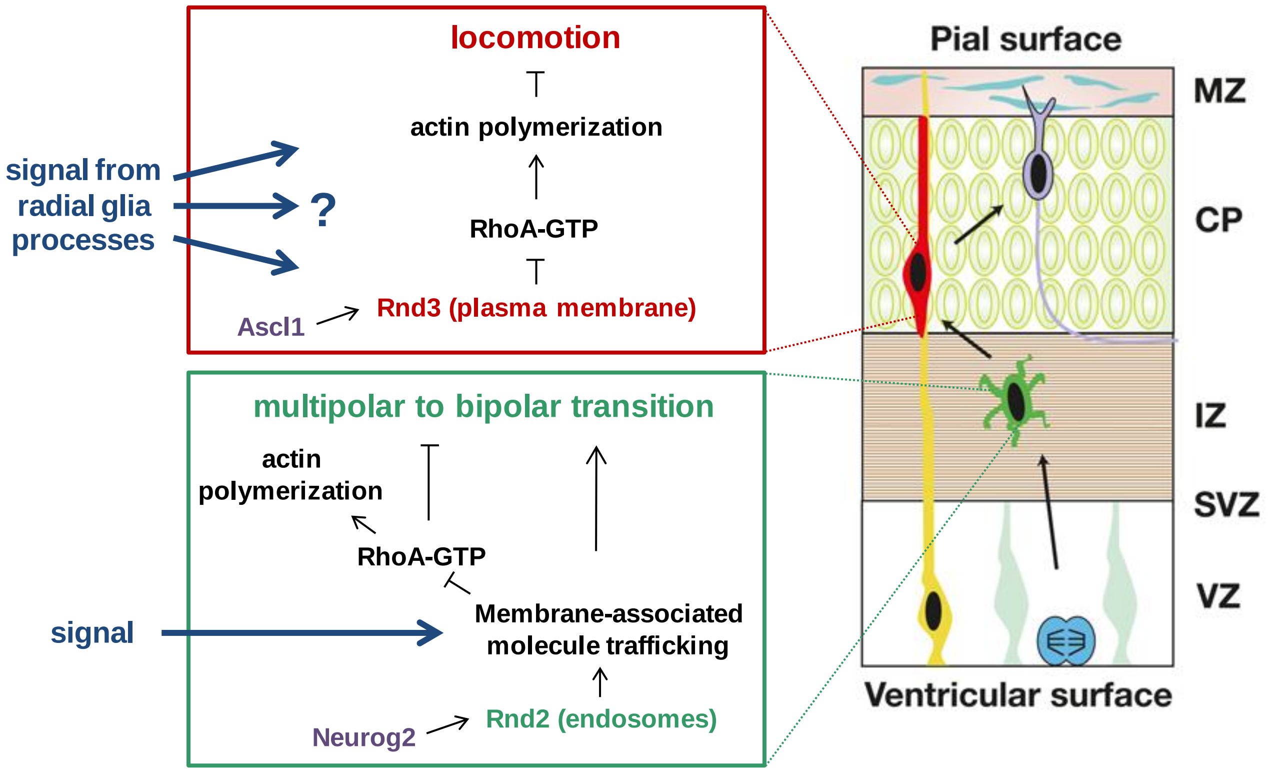

Figure S1, Related to Figure 1. Identification of *Rnd3* as a Novel Direct Target of Ascl1

(A) *Ascl1* conditional mutant allele. *Ascl1* was targeted by homologous recombination to generate the allele *Ascl1^{flox}*. This allele contains one loxP site located at the start codon of *Ascl1*, a second loxP site located after the polyA signal at the 3' end of *Ascl1* transcript, immediately followed by a Venus cassette. A neo selection gene flanked by FRT sites, was inserted in 3' of *Ascl1*, as illustrated, but the FRT sites are not functional in vivo. Upon cre recombination, the entire coding sequence and 3' UTR of *Ascl1* is deleted and the expression of the Venus cassette is now under the control of the *Ascl1* upstream regulatory elements. However, the level of expression of the Venus reporter gene is below detection.

(B and C) Expression of Ascl1 protein in the cerebral cortex of *Ascl1$^{flox/flox}$* embryos at E14.5 (B) and E17.5 (C).

(D) Expression of *Rnd2* and *Rnd3* was analyzed by RNA in situ hybridization on coronal sections of E16.5 telencephalon in *Ascl1-/-* and *Neurog2-/-* embryos. *Rnd2* expression was not affected in *Ascl1-/-* embryos and *Rnd3* expression was not significantly affected in *Neurog2-/-* embryos.

(E) The ventral telencephalon of E10.5 wild-type embryos was electroporated with a *Ascl1* expression construct and the electroporated tissue harvested 18 hrs later and analyzed by Affymetrix expression microarrays to identify genes regulated by gain-of-function (GOF) of *Mash1*. The dorsal telencephalon of wild-type, and *Ascl1-/-* E13.5 embryos were analyzed by Affymetrix expression microarrays to identify genes regulated by loss-of-function (LOF) of *Ascl1*. *Rnd3* transcripts were up-regulated in *Ascl1* overexpressing embryos and down-regulated in *Ascl1-/-* embryos.

(F) Localization of Ascl1-type E boxes in the vicinity of the *Rnd3* locus that are conserved in the mouse, rat and human genomes. Chromatin immunoprecipitation (ChIP) analysis of the binding of Ascl1 to *Rnd3*-associated conserved elements in chromatin prepared from the ventral telencephalon of E12.5 wild-type embryos. The control experiment used the same procedure without antibody. Four *Rnd3* enhancers bound Ascl1 significantly, however the level of binding of E11 and E14 was low and only E1 and E5 were further analyzed. Student's *t*-test; *$p<0.05$, ***$p<0.001$; mean ± SEM of triplicate quantifications from at least two immunoprecipitations.

Scale bar represents 50 µm (B and C).

Figure S2, Related to Figure 2. *Rnd3* Knockdown Affects Neuronal Migration Independently of Its Role in Progenitor Proliferation and It Does Not Affect Cell Death or the Morphology of Radial Glial Processes

(A) Western blot of P19 cells transfected with constructs as indicated and probed with anti-flag and anti-actin antibodies. Both *Rnd3* shRNAs efficiently silenced *Rnd3* expression. Expression of actin was used as a loading control.

(B) Ex vivo electroporation of E14.5 cortices with *Rnd3#1* shRNA or *Rnd3#2* shRNA followed by 4 days of slice culture resulted in similar migration defects. One way ANOVA followed by a Fisher PLSD post hoc test; **p<0.01, ***p<0.001 compared to empty construct; #p<0.05, ##p<0.01, ###p<0.001 compared to ctrl shRNA; mean ± SEM; n=8-9 slices.

(C) Ex vivo electroporation of *Rnd3* shRNA followed by 2 days of slice culture did not change the fraction of electroporated cells labelled with an antibody against activated caspase 3 indicating that *Rnd3* silencing does not induce apoptotic cell death. mean ± SEM, n=9-10 sections in each experiment.

(D) *Rnd3*-knockdown followed by 2 days of slice culture increased significantly the fraction of electroporated cells having incorpored BrdU during a 2 hr exposure. Student's *t*-test; **p<0.01; mean ± SEM; n=9 slices.

(E) *Rnd3*-knockdown in vivo at E14.5 did not affect the morphology of radial glial processes analyzed 3 days later for nestin expression.

(F) Ex vivo electroporated E14.5 brains were maintained in culture for 4 days in the continuous presence of BrdU and the migration of GFP-positive and BrdU-negative cells (cells postmitotic at the time of the electroporation) was quantified. The migration defects of these cells are similar to those of all electroporated cells (Figure S2B). Student's *t*-test, **p<0.05, mean ± SEM, n=9 slices.

(G) Ex vivo electroporation of the cortex of E14.5 *Nex-Cre* mice with pBS/U6-ploxPNeo-*Rnd3* shRNA (loxP-*Rnd3* shRNA) or pBS/U6-ploxPNeo-scr shRNA (loxP-ctrl shRNA). pBS/U6-ploxPNeo-*Rnd3* shRNA was used for conditional expression of *Rnd3* shRNA. Activation of *Rnd3* shRNA expression by *Nex-Cre* in postmitotic neurons resulted in similar defects as expression of *Rnd3* shRNA in all electroporated cells. Student's *t*-test; ***p<0.001; mean ± SEM; n=7-9 slices.

Scale bars represent 20 µm (C and D), 50 µm (E) and 100 µm (B, F, and G).

Figure S3, Related to Figures 2 and 4. Both *Rnd2* and *Rnd3* Silencing Produce Migration Defects that Persist at Postnatal Stages

(A) Cortices were electroporated in utero at E14.5 and analyzed at postnatal day 2 (P2). Electroporated cortices were subdivided into 9 bins. A fraction of *Rnd2*, *Rnd3* and *Rnd2+Rnd3*-silenced neurons failed to migrate and remained near the ventricle at P2 (bin 9). One way ANOVA followed by a Fisher PLSD post hoc test; *p<0.05,***p<0.001 compared to ctrl shRNA; #p<0.05, ##p<0.01 compared to *Rnd3* shRNA; + p<0.05 compared to *Rnd2* shRNA.

(B) Cortices were electroporated in utero at E14.5 with GFP and different shRNAs constructs as indicated and analyzed three days later. One way ANOVA followed by a Fisher PLSD post hoc test; **p<0.01, ***p<0.001 compared to control (ctrl) shRNA; ##p<0.01 compared to *Rnd3* shRNA; +p<0.05, ++p<0.01 compared to *Rnd2* shRNA. Scale bars represent 150 µm (B) and 200 µm (A).

Figure S4, Related to Figure 2. Overexpression of *Rnd3* Rescues the Radial Migration Defects of *Ascl1*-Mutant Cortical Neurons

(A) In utero electroporation of E14.5 embryos homozygous for a conditional null mutant allele of *Ascl1* ($Ascl1^{flox/flox}$) with Cre to delete *Ascl1* acutely prevented many electroporated neurons from leaving the VZ/SVZ and IZ and reaching the CP. Co-electroporation of *Rnd3* rescued the migration defects caused by *Ascl1* deletion while co-electroporation of Rnd2 did not have an effect. One way ANOVA followed by a Fisher PLSD post hoc test; *p<0.05, **p<0.01, ***p<0.001; mean ± SEM.

(B) Overexpression of *Rnd3* at a moderate (pCMV-Rnd3) or high (pClG-Rnd3) level by in utero electroporation at E14.5. *Rnd3* overexpression at a moderate level had no apparent effect on the morphology or the migration of cortical neurons while overexpression at a high level resulted in reduced migration of electroporated cells. Student's *t*-test; ***p<0.001 compared to control.

Scale bars represent 150 µm (A and B).

Figure S5, Related to Figure 5. *RhoA* Knockdown Rescued the Radial Migration Defects of *Ascl1*-Mutant Cortical Neurons

(A) Western blot analysis of P19 cells demonstrating that the *RhoA* shRNA construct used efficiently silenced *RhoA* expression.

(B) *RhoA* silencing affects neuronal migration in the cortex and overexpression of human RhoA (resistant to *RhoA* shRNA) fully rescued these defects.

(C) Co-electroporation of *RhoA* shRNA rescued the migration defects of *Ascl1* mutant neurons in Cre-electroporated $Ascl1^{flox/flox}$ embryos. (B and C) One way ANOVA followed by a Fisher PLSD

post hoc test; *p<0.05, **p<0.01; ***p<0.001; mean ± SEM. Scale bars represent 150 µm (B and C).

Figure S6, Related to Figure 5. Rnd3 Antagonizes *RhoA* Signaling in Migrating Cortical Neurons by Stimulating the RhoGAP Activity of p190 and Not by Inhibiting ROCKI

(A) Western blot analysis of P19 cells demonstrating that mutated versions of *Rnd3* encoding wild-type Rnd3 (marked *Rnd3**) are resistant to *Rnd3* shRNA silencing.

(B) Lysates of COS7 cells expressing Myc-Δp190B and FLAG-Rnd3 or FLAG-Rnd3^{T55V} or FLAG-Rnd2 or FLAG-Rnd2^{T39V} were immunoprecipitated with anti-FLAG antibody. The precipitates were separated by SDS-PAGE and analyzed by immunoblotting (IB). Mutation of the effector domain of the Rnds abrogates their interaction with p190RhoGAP-B.

(C and D) Expression of resistant *Rnd3** fully rescued the neuronal migration and morphology defects resulting from *Rnd3* silencing while Rnd3^{T55V}* was inactive. In contrast, *Rnd3*$^{T173R/V192R}$* was as active as *Rnd3** at rescuing the migration and morphology of *Rnd3*-silenced neurons. (C) One way ANOVA followed by a Fisher PLSD post hoc test; *p<0.05, **p<0.01; ***p<0.001; mean ± SEM. (D) n>500 cells from 3 different brains; Student's *t*-test; **p<0.01, ***p<0.001 compared to control shRNA.

(E) Western blot analysis of P19 cells demonstrating that Rnd2^{T39V}* is resistant to *Rnd2* shRNA silencing.

(F) Expression of *Rnd2*^{T39V}* rescued the neuronal morphology defects resulting from *Rnd2* silencing like *Rnd2**. One way ANOVA followed by a Fisher PLSD post hoc test; *p<0.05, **p<0.01, ***p<0.001; mean ± SEM. Scale bars represent 150 µm (C, F).

Figure S7, Related to Figure 7. Rnd3 Is Associated with Both the Plasma Membrane and Endosomes While Rnd2 Is Only Associated with Endosomes

(A) E14.5 cortical neurons cultivated for 2 days and co-labelled with antibodies against Rnd2 or Rnd3 and N-cadherin, a protein associated with the plasma membrane, or with markers of the different endosomal compartments as indicated. Rnd3 co-localized with N-cadherin, EEA1 and transferrin receptor (TfR) whereas Rnd2 co-localized only with EEA1. Scale bar represents 5 µm (A).

(B) Western blot analysis of P19 cells demonstrating that the mutated versions of Rnd3, Rnd3^{C241S}* and Rnd3$^{All\ A}$*are resistant to silencing by *Rnd3* shRNA.

Figure S8, Related to Figure 8.

(A) Alignment of the sequences of Rnd2 and Rnd3 proteins and sequence of Rnd2^{Rnd3Cter} protein.

(B) Model of the roles of the Neurog2-Rnd2 and Ascl1-Rnd3 pathways in the regulation of cortical neuronal migration. Neurog2 and Ascl1 regulate distinct steps of cortical neuron migration, i.e. multipolar to bipolar transition in the IZ for Neurog2 and locomotion in the CP for Ascl1, through Rnd-mediated inhibition of RhoA signaling in different compartments of the cell. Ascl1 induces expression of plasma membrane-localized Rnd3, which inhibits RhoA and induces F-actin depolymerization, resulting in stabilization and attachment of the leading process to a radial glial fiber. This in turn allows nucleokinesis and locomotion to proceed. Neurog2 induces early endosome-localized Rnd2, which may regulate the trafficking of membrane-associated molecules that promote neuronal polarization and extension of the leading process at the multipolar to bipolar transition. Rnd2-mediated inhibition of RhoA but not F-actin depolymerization is necessary for this process. Multiple extracellular signals may regulate these two pathways at different levels to coordinate neuronal migration.

SUPPLEMENTAL EXPERIMENTAL PROCEDURES

Generation and Genotyping of *Ascl1*$^{flox/flox}$ Mice

The generation of *Ascl1*$^{floxed/floxed}$ mice is described in Figure S1A. Genotyping of *Ascl1* wild-type allele was performed with the following primers : forward, 5'-CTACTGTCCAAACGCAAAGTGG-3' and reverse 5'- GCTCCCACAATCCTCGTAAAGA-3'. Genotyping of *Ascl1* conditional mutant allele was performed with the same forward primer as for the wild-type allele and the following reverse primer 5'-TAGACGTTGTGGCTGTTGTAGT-3'. PCR conditions were 35 cycles of 94°C/30 sec; 64°C/30 sec; 72°C/1 min 30 sec.

Plasmid Constructs

Rnd3, Rnd3^{T55V}, Rnd3$^{T173R/V192R}$, Rnd3^{C241S}, Rnd3$^{All\ A}$ subcloned into the pCMV-Flag vector were kind gifts from Dr Anne Ridley, pClG2-Centrin2-Venus construct from Dr. Mary E. Hatten, RhoA probe for FRET experiments (pRaichu1298x and pRaichu1293x) from Michiyuki Matsuda, EGFP-UTRCH-ABD construct from William M. Bement and pcDNA-cofilin^{S3A} from Iryna M. Ethell. pcDNA-hRhoA was obtained from Missouri S&T cDNA Resource Center and pAcGFP1-Mem vector was purchased from Clontech. The Cre expression plasmid pCIG2-Cre as well as the pNeuroD1-Rnd2*-IRES-GFP expression plasmid have been described previously (Heng et al., 2008).

The full length coding sequence for mouse *Rnd2* was cloned by PCR using pCR-Rnd2 as template and then inserted into the EcoRI/HindIII sites of the pCMV-Flag vector to generate pCMV-Flag-Rnd2. Flag-Rnd3 was cloned by PCR using pCMV-Flag-Rnd3 as a template into XhoI/PstI sites of the pClG2 vector to generate pClG2-Flag-Rnd3. Flag-Rnd3 blunted fragment from pClG2-Flag-Rnd3 was cloned into blunted EcoRI sites of pNeuroD1-IRES-GFP expression plasmid to generate pNeuroD1-Rnd3-IRES-GFP.

To generate expression constructs harboring silent point mutations in the sequence recognized by *Rnd3* shRNA (*Rnd3* shRNA#2), site-directed mutagenesis was performed using QuickChange II Site-Directed Mutagenesis Kit (Stratagene) on pCMV-Rnd3, pCMV-Rnd3^{T55V}, pCMV-Rnd3^{C241S} and pCMV- Rnd3$^{All\ A}$ plasmids using the following primers: forward, 5'-CCAAGCAGATCGGAGCAGCCACTTATATTGAGTGCTCAGCTTTAC-3' and reverse, 5'-GTAAAGCTGAGCACTCAATATAAGTGGCTGCTCCGATCTGCTTGG-3'. The underlined nucleotide residues identify silent mutations on nt522(C→T), nt525(A→T) and nt528(A→G) of the Rnd3 cDNA sequence. *Rnd3** was used to indicate the mutated *Rnd3* cDNA. To generate pCMV-Rnd3$^{T173R/V192R}$*, the following primers were used: forward, 5'-ATGGCCAAGCAGATCGGAGCAGCCAGATATATTGAGTGCTCAGCTTTACAGTCAG-3'

and reverse 5'-CTGACTGTAAAGCTGAGCACTCAATATATCTGGCTGCTCCGATCTGCTTGGCCAT-3'.

To generate pND-Rnd2^{T39V}, pCMV-F-Rnd2^{T39V} and pClG2-F-Rnd2^{T39V}, the following primers were used: forward, 5'-CCCGGGAGTTATGTCCCCGTGGTGTTTGAGAACTACAC-3' and reverse 5'-GTGTAGTTCTCAAACACCACGGGGACATAACTCCCGGG-3'.

Rnd2^{Rnd3Cter}, generated by three successive PCR, was inserted into the EcoRI/HindIII sites of the pCMV-Flag vector to generate pCMV-Flag-Rnd2^{Rnd3Cter}.

For luciferase reporter assays, *Rnd3* E1 and E5 enhancers were cloned from mouse genomic DNA by PCR into the SalI/NheI sites of the luciferase reporter vector p-βglob-Luciferase (Heng et al., 2008). Mutations were generated using a QuikChange II Site-Directed Mutagenesis kit (Stratagene). To generate a mutation in the Ascl1 binding motif of the E1 element (designated as E1^{mut}), the following primers were used: forward, 5'-CAGCGAGGGGTAGTAGCCTGCTGGCCGGGCAG-3' and reverse, 5'-CTGCCCGGCCAGCAGGCTACTACCCCTCGCTG-3'. To generate a mutation in the Ascl1 binding motif of the E5 element (designated as E5^{mut}), the following primers were used: forward, 5'-GCTCTTACTGAATGACTGTACCCTCAGCTGCAGCCAGTCCAA-3' and reverse, 5'-TTGGACTGGCTGCAGCTGAGGGTACAGTCATTCAGTAAGAGC-3'.

Rnd3 E1 and E5 enhancers were also cloned as 3' enhancer elements into the SalI/ApaI sites of a LacZ reporter vector harbouring a minimal *β*-globin promoter for the subsequent generation of transgenic reporter mice as previously described (Heng et al., 2008).

Rnd2 shRNA, *Rnd3* shRNA#1 and *Rnd3* shRNA#2 were obtained by cloning the following sequences into the short hairpin RNA vector, pCA-b-EGFPm5 silencer 3, (generous gift from Dr Vermeren): Rnd2, 5'-GGGCGAGATGCATAAGGAT-3' (Ambion, ID 65909); Rnd3#1, 5'-GCACATTAGTGGAACTCTC-3' (Ambion, ID 166122); Rnd3#2, 5'-GCAGCCACTTACATAGAAT-3' (Ambion, ID 166123). Control (ctrl) shRNA construct was generated by cloning into pCA-b-EGFPm5 silencer 3 a 19 bp scrambled sequence (5'-TACGCGCATAAGATTAGGG-3') with no significant homology to any known gene sequence from mouse or human as described previously (Kawauchi et al., 2006). For FRET experiments, EGFP in ctrl shRNA, *Rnd2* shRNA and *Rnd3* shRNA plasmids was replaced by RFP. To allow conditional expression of ctrl shRNA and *Rnd3#2* shRNA using the Cre-LoxP system, sequences indicated previously were cloned into pBS/U6-ploxPneo vector (generous gift from Dr Deng) as previously described (Shukla et al., 2007). The resulting constructs were named loxP-ctrl shRNA and loxP-*Rnd3* shRNA. *Ascl1* shRNA (GI546876, 5'-AGAAGATGAGCAAGGTGGAGACGCTGCGC-3') and *RhoA* shRNA (GI564020, 5'-

CAAGAAGGACCTTCGGAATGACGAGCACA-3') were purchased from Origene. The non targeting shRNA pGFP-V-RS plasmid served as control (TR30007, Origene).

All of the above mentioned constructs were fully sequenced verified before their use in experiments.

In Utero Electroporation and Tissue Processing

In utero electroporation was performed as described previously (Nguyen et al., 2006) with minor modifications. Briefly, uteri of anaesthetized timed-pregnant mothers (14 days) with isoflurane in oxygen carrier were exposed through a 1 centimeter incision in the ventral peritoneum. The pregnant mouse was injected before the surgery with buprenorphine (Vetergesic; Alstoe Ltd). Embryos were carefully lifted using ring forceps through the incision and placed on humidified gauze pads. DNA was prepared in endotoxin-free conditions (Qiagen) and was injected at the following concentrations: shRNA constructs, 1 µg/µl; rescue constructs, 1 µg/µl; pClG2-Cre, 1 µg/µl; pClG2-Centrin2-Venus, 2 µg/µl; RhoA probe, 0.25 µg/µl. Plasmid DNA solution mixed with 0.05% Fast Green (Sigma) was injected through the uterine wall into the telencephalic vesicle using pulled borosilicate needles (Harvard Apparatus) and a Femtojet microinjector (Eppendorf). Five electrical pulses were applied at 30V (50 msec duration) across the uterine wall at 1 sec intervals using 5 mm platinum electrodes (Tweezertrode 45-0489, BTX, Harvard Apparatus) connected to an electroporator (ECM830, BTX). The uterine horns were then replaced in the abdominal cavity and the abdomen wall and skin were sutured using surgical needle and thread. The pregnant mouse was warmed on heating pad until it woke up. The whole procedure was complete within 30 min. Three days following the surgery, pregnant mice were sacrificed by neck dislocation and embryos were processed for tissue analyses. Embryonic brains were fixed in 4% PFA overnight and then placed in 20% sucrose/PBS overnight. P2 brains were dissected from anesthetized pups subjected to intracardial perfusion of 0.9% NaCl, followed by 4% PFA in PBS. Then brains were postfixed in 4% PFA and fixed samples were cryoprotected overnight in 20% sucrose in PBS at 4°C. Embryonic and postnatal brains were then embedded in OCT Compound and frozen before sectioning using a cryostat.

The different subregions of the cerebral cortex (VZ/SVZ, IZ and CP) were identified based on cell density and visualized with TOTO-3 iodide nuclear staining (Invitrogen). All images were acquired with a laser scanning confocal microscope (Radiance 2100, BioRad). For migration experiments, six sections were analyzed for each condition from three embryos from two or three litters obtained in parallel experiments. Cell counts were performed using MetaMorph software (Molecular Devices).

A statistical analysis was performed using either unpaired two-tailed Student's t-test between control and experimental condition, or one-way ANOVA followed by a PLSD Fisher post hoc test for multiple comparisons (StatView software, version 5).

Ex Vivo Cortical Electroporation and Dissociated Cell Cultures

Ex vivo electroporation was performed on injected mouse embryos' heads similarly to in vivo electroporation. The electrical parameters were the following: 50V, 50 msec length, 5 pulses, 1 sec interval. Following electroporation, brains were dissected in L15 (PAA Laboratories) and transferred into liquid 3% low melting agarose (Sigma) and incubated on ice for 1 hr. Embedded brains were cut coronally (300 µm) with a vibratome (Leica), and slices were transferred onto sterilized culture plate inserts (0.4-µm pore size; Millicell-CM, Millipore) and cultivated in complete Neurobasal containing Neurobasal medium (Invitrogen) supplemented with 1% B27, 1% N2, 1% glutamine, 1% penicillin/streptomycin and 1% fungizone. Slices were cultured for up to 4 days, culture medium was renewed every day and slices were fixed with 4% PFA and processed for immunohistochemistry. For BrdU incorporation study, slices were incubated after 2 DIV with 10 µM BrdU (Sigma) for the last 2 hr of culture. To study the migration of postmitotic neurons (GFP^+/$BrdU^-$ cells), culture medium was renewed every day with 10 µM BrdU.

For dissociated cell cultures, slices were cultivated one day following ex vivo electroporation and the electroporated part of the cortex was microdissected under a GFP binocular. Tissues were pooled in complete Neurobasal medium containing 100µg/ml DNAse (Sigma) and triturated with Pasteur pipettes. Dissociated cortical cells were seeded on poly-Dlysine (10 µg/ml, Sigma) and laminin (10 µg/ml, Sigma) coated wells (Lab-Tek, Chamber Slide™, 4 well Permanox® slides) and cultured in complete Neurobasal medium. After 2 DIV, neurite length and number were measured using ImageJ (Rasband, W.S., ImageJ, U.S. National Institutes of Health, Bethesda, Maryland, USA, http://rsb.info.nih.gov/ij/).

Live Imaging

One day after ex vivo electroporation, GFP was imaged in live brain slices using 900nm multiphoton excitation (Spectraphysics DeepSee) with a Leica SP5 confocal scanner on a DM6000 CFS upright microscope. A 10x,0.4NA (dry) lens was used and reflected excitation collected with a non descanned PMT through a 525/50 filter (Chroma).

Fluorescence Resonance Energy Transfer (FRET)

Samples for analysis of FRET by acceptor photobleaching were imaged using a Zeiss LSM 510 META laser scanning confocal microscope and a 25 x NA 1.4 Ph2 or 63 x Plan Apochromat NA 1.4 Ph3 oil objective as specified. The CFP and YFP channels were excited using the 440 nm diode laser and the 514 nm argon line respectively. The two emission channels were split using a

545 nm dichroic mirror, which was followed by a 475-525 nm bandpass filter for CFP and a 530 nm longpass filter for YFP (Chroma). Pinholes were opened to give a depth of focus of 2 µm for each channel. Scanning was performed on a sequential line-by-line basis for each channel. The gain for each channel was set to approximately 75 % of dynamic range (12-bit, 4096 grey levels) and offsets set such that backgrounds were zero. Time-lapse mode was used to collect one pre-bleach image for each channel followed by bleaching with a minimum of 50 iterations of the 514 nm argon laser line at maximum power (to bleach YFP). A second post-bleach image was then collected for each channel. Control non-bleached areas were acquired for all samples in the same field of view as bleached cells to confirm specificity of FRET detection. Pre- and post-bleach CFP and YFP images were then imported into Image J for processing. Briefly, images were smoothed using a 3 x 3 box mean filter, background subtracted and post-bleach images fade compensated. A FRET efficiency ratio map over the whole cell was calculated using the following formula: $(CFP_{postbleach}-CFP_{prebleach})/CFP_{postbleach}$. Ratio values were then extracted from pixels falling inside the bleach region as well as an equally sized region outside of the bleach region and the mean ratio determined for each region and plotted on a histogram. The non-bleach ratio was then subtracted from the bleach region ratio to give a final value for the FRET efficiency ratio. Data from images were used only if YFP bleaching efficiency was greater than 70 %.

Cell Cultures

Primary cortical neuron cultures were prepared from E14.5 mouse cortices. Dissected cortices were pooled in complete Neurobasal medium and triturated with Pasteur pipettes. Dissociated cortical cells were cultured as just described.

HEK 293 cells and mouse embryocarcinoma cell line P19 were grown in DMEM supplemented with 10% fetal bovine serum (FBS), 2 mM glutamine and 1% penicillin/streptomycin and transfected with lipofectamine according to the manufacturer's protocol (Invitrogen). Primary cortical neurons, HEK and P19 cells were cultured in humidified incubator at 37°C under 5% CO_2 atmosphere.

Western Blotting

After extraction of total proteins or nuclear proteins for Ascl1 in the presence of protease and phosphatase inhibitors (Sigma), proteins were separated on 4-12% gradient gels using the XCell Surelock Mini-Cell and the NuPAGE® MES SDS Running Buffer (Invitrogen). Proteins were then transferred to nitrocellulose membranes (Amersham) using the XCell II Blot Module and the NuPAGE® Transfer Buffer (Invitrogen). Membranes were blocked for 1 h in 5% non fat milk in Tris-buffered saline (TBS) containing 0.05% Tween 20 (T-TBS). Membranes were then

incubated overnight with the following primary antibodies diluted in the blocking solution: rabbit anti-actin (1/1000, Sigma), mouse anti-Ascl1 (1/500, R&D systems), mouse anti-Flag (1/1000, Sigma), rabbit anti RhoA (1/200, Santa Cruz), mouse anti-Rnd3 (1/1000, Upstate). After washing in T-TBS, membranes were incubated for 1 h at room temperature with peroxidase-labelled secondary antibody. The immunoreactive bands were visualized using enhanced chemiluminescent detection reagents according to the manufacturer's instructions (ThermoScientific).

In Silico Search for Ascl1 Binding Sites and Chromatin Immunoprecipitation Assays

Conserved Ascl1 binding sites across mouse, rat and human genomes within the *Rnd3* gene locus were identified using the UC Santa Cruz genome browser (http://genome.ucsc.edu/cgi-bin/hgGateway).

Chromatin immunoprecipitation (ChIP) assays were performed as previously described (Castro et al., 2006) with chromatin prepared from E12.5 ventral telencephalon and with a monoclonal mouse anti-Ascl1 antibody or without antibody as a negative control. Immunoprecipitated chromatin was quantified using the Real Time PCR (AB Applied Biosystems) and a SYBR-Green based kit for quantitative PCR (iQ Supermix, BioRad). Quantities of immunoprecipitated chromatin were calculated by comparison to a standard curve generated by serial dilutions of input chromatin. Plotted data represent the mean of two independent assays and three independent amplifications. The primers used for amplification of the *Rnd3* E1 and E5 enhancers are the following:

E1 Fwd: 5'-TGCTGCTCTTGCTTTGTCTC-3'

E1 Rev: 5'-CGTTCCCTGCTGCTCTAAT-3'

E5 Fwd: 5'-AATTACTCAGCTTGGGCACAG-3'

E5 Rev: 5'-CATTTCCTCCTACGGCTCAT-3'

Luciferase Assays

For luciferase assays, P19 cell transfections were performed in triplicate in 24-well plates by using Lipofectamine 2000. Each well was transfected with 250 ng appropriated expression plasmids, 125 ng luciferase reporter plasmid, and 250 ng CMV-β-gal plasmid as internal control. Cells were lysed 24 hr after transfection (Passive Lysis Buffer, Promega), and extracts were assayed for luciferase and β-gal activities.

COS7 Cell Electroporation and Immunoprecipitation

pCMV-Myc-Δp190B (encoding amino acids 382-1007 of p190RhoGAP-B) was a gift from Steen Hansen (Wennerberg et al., 2003). COS7 cells grown in DMEM containing 10% FBS and penicillin/streptomycin were transfected by electroporation in 250 μl of ice-cold electroporation

buffer (120 mM KCl, 10 mM K_2PO_4/KH_2PO_4, pH 7.6, 25 mM Hepes, pH 7.6, 2 mM $MgCl_2$, and 0.5% Ficoll). DNA (5 μg per cDNA construct) was electroporated into cells at 250 V and 960 mF using 0.4-cm Gene Pulser Cuvettes (Bio-Rad). The cells were then grown in normal medium. After 24 h, transfected COS7 cells were lysed in lysis buffer (1% Triton X-100, 20 mM Tris–HCl, pH 8, 130 mM NaCl, 1 mM dithiothreitol (DTT), 10 mM sodium fluoride, complete EDTA-free protease inhibitor cocktail (Roche), phosphatase inhibitor cocktails (set II+IV, Calbiochem)). After removal of insoluble material by centrifugation at 13,000g at 4°C for 10 min, soluble proteins in the cell lysates were precleared and subsequently incubated with mouse anti-FLAG M2 antibody covalently linked to agarose beads (Sigma) for 2 h at 4°C. Beads were washed with lysis buffer (containing 260 mM NaCl), then the bound proteins eluted in Laemmli sample buffer, resolved by SDS–PAGE and analysed by immunoblotting. Rabbit anti-myc antibody (Santa Cruz) and rabbit anti-FLAG antibody (Sigma) were used for detection of myc-tagged and FLAG-tagged proteins respectively.

SUPPLEMENTAL REFERENCES

Castro, D. S., Skowronska-Krawczyk, D., Armant, O., Donaldson, I. J., Parras, C., Hunt, C., Critchley, J., Nguyen, L., Gossler, A., Gottgens, B., *et al.* (2006). Proneural bHLH and Brn proteins co-regulate a neurogenic programme through cooperative binding to a conserved DNA motif. Dev Cell *11*, 831-844.

Heng, J. I.-T., Nguyen, L., Castro, D. S., Zimmer, C., Wildner, H., Armant, O., Skowronska-Krawczyk, D., Bedogni, F., Matter, J. M., Hevner, R. F., and Guillemot, F. (2008). Neurogenin2 controls cortical neuron migration through regulation of Rnd2. Nature *455*, 114-118.

Kawauchi, T., Chihama, K., Nabeshima, Y., and Hoshino, M. (2006). Cdk5 phosphorylates and stabilizes p27kip1 contributing to actin organization and cortical neuronal migration. Nat Cell Biol *8*, 17-26.

Nguyen, L., Besson, A., Heng, J. I., Schuurmans, C., Teboul, L., Parras, C., Philpott, A., Roberts, J. M., and Guillemot, F. (2006). p27kip1 independently promotes neuronal differentiation and migration in the cerebral cortex. Genes Dev *20*, 1511-1524.

Shukla, V., Coumoul, X., and Deng, C. X. (2007). RNAi-based conditional gene knockdown in mice using a U6 promoter driven vector. Int J Biol Sci *3*, 91-99.

Wennerberg, K., Forget, M. A., Ellerbroek, S. M., Arthur, W. T., Burridge, K., Settleman, J., Der, C. J., and Hansen, S. H. (2003). Rnd proteins function as RhoA antagonists by activating p190 RhoGAP. Curr Biol *13*, 1106-1115.
